# A Systematic Review of Community-based Interventions to Promote Physical Activity or Reduce Sedentary Behavior among Adults in Low- and Middle-Income Countries

**DOI:** 10.1007/s13679-026-00741-4

**Published:** 2026-07-22

**Authors:** Madhur Verma, Madhu Gupta, Gursimer Jeet, Hans Bosma, Annemarie Koster

**Affiliations:** 1 Department of Social Medicine, CAPHRI Care and Public Health Research Institute, Maastricht University, Maastricht, The Netherlands; 2 Department of Community and Family Medicine, All India Institute of Medical Sciences, Bathinda, Punjab 151001 India; 3 Department of Community Medicine and School of Public Health, Post Graduate Institute of Medical Education and Research, Chandigarh, 160011 India; 4 Consultant- Health Impact Assessment, British Columbia, V6M1T5 Canada

**Keywords:** Behavioral risk factors, Community-based interventions, Low-middle-income countries, Physical activity, Sedentary behavior

## Abstract

**Purpose of Review:**

Community-based interventions (CBIs) are widely used to address behavioral risk factors for non-communicable diseases, yet evidence of their effectiveness in low- and middle-income countries (LMICs) remains limited. This study systematically reviewed evidence on the effectiveness of CBIs promoting physical activity (PA) and/or reducing sedentary behavior (SB) among community-dwelling adults in LMICs. We searched PubMed, Embase, Scopus, and the Cochrane Library (2000–2024) for CBIs in LMICs. Primary outcomes were changes in PA and/or SB. Descriptive and graphical depictions were used to draw inferences.

**Recent Findings:**

We selected 24 studies (11 RCTs) from 15,396 for review. Most studies (*n* = 16) reported significant improvements in PA outcomes. Changes were observed in Metabolic Equivalents (+ 100 - +2,700), moderate-to-vigorous PA (+ 7 to + 60 min/week), and steps/day (+ 3,000). Broadly, interventions were delivered using digital/technology format (*n* = 10), peer-led/community health worker-facilitated (*n* = 8), and Environmental restructuring (*n* = 6), with the digital interventions depicting the most consistent significant improvements. Two studies directly measured SB as a primary outcome. Short- (≤ 6 months; *n* = 11) and medium-duration (6–12 months; *n* = 4) interventions more frequently reported significant PA improvements than long-duration interventions (> 12 months; *n* = 9). PA/SB-focused CBI demonstrated proportions of significant effects similar to those of broader lifestyle interventions (88.9% and 53.3%, respectively).

**Summary:**

CBIs can be effective in improving PA in LMICs, although evidence is heterogeneous and SB is less addressed. Digital interventions show more consistent positive signals, but high heterogeneity in studies precludes firm conclusions. The impact of duration on effectiveness looks counterintuitive but underscores the need for more rigorous evaluation of longer-duration interventions and SB outcomes.

**PROSPERO registration number:** CRD42024579461.

**Supplementary Information:**

The online version contains supplementary material available at 10.1007/s13679-026-00741-4.

## Introduction

Life expectancy has increased globally due to socioeconomic improvements, better diets, enhanced public health measures, and advancements in the quality of medical care [1]. While Infectious diseases have declined, non-communicable diseases (NCDs) now drive most morbidity and mortality [2], straining the health systems through chronic care needs. The natural history of NCDs is influenced by non-modifiable (e.g., age, genetics) and modifiable risk factors, with behavioral determinants (e.g., physical inactivity, sedentary behavior, unhealthy diet, etc.) forming a critical subset of modifiable risks. Of these, physical activity (PA) and sedentary behavior (SB) are among the most crucial. Regular moderate or vigorous PA can prevent chronic diseases, maintain cardiorespiratory fitness, and reduce all-cause mortality, whereas high levels of SB are independently linked with adverse health outcomes that PA cannot fully offset [3, 4]. In response, the World Health Organization (WHO) launched a global action plan to reduce physical inactivity by 10% by 2025 and 15% by 2030 [5]. The plan recommends that all adults engage in moderate-intensity (150–300 min/week) or vigorous-intensity PA (75–150 min/week), or an equivalent combination of both, while reducing SB [6]. 

Despite the known health benefits, the prevalence of physical inactivity and SB remains widespread [5]. A multi-country analysis estimated the global age-standardized prevalence of insufficient physical activity to be 31.3% in 2022 [7]. Approximately 31% of individuals aged ≥ 15 years are physically inactive, and this causes between 3.2 and 5 million deaths globally per year [6, 8]. Although high-income countries (HICs) have nearly twice the prevalence (36.8%, 35.0–38.0%) compared to low- and middle-income countries (LMICs) (16.2%, 14.2–17.9%) [7], levels of physical inactivity in LMICs are increasing rapidly due to urbanization and declining active transport [9]. This growing inactivity burden makes LMICs increasingly vulnerable to NCD–related impacts [6]. These alarming epidemiological trends will negatively impact the attainment of global targets and multiple SDGs, and necessitate urgent interventions to improve PA levels, especially in LMICs with already overwhelmed health systems [10]. 

Evidence from HICs shows multiple effective PA interventions [11], but long-term benefits depend on implementation beyond clinical settings. Community-based interventions (CBIs) are preferred for their real-world reach, population impact, and ability to address behavioral, social, and environmental determinants through participatory and multi-sectoral approaches [12]. The International Society for Physical Activity and Health (ISPAH) identifies CBIs as one of eight key global investments to reduce the prevalence of insufficient PA [13]. Previous systematic reviews from LMICs provide important contemporary evidence [14–16]. A closely related systematic review and meta-analysis by Shrestha et al. has already estimated the overall effectiveness of PA promotion interventions in LMICs and provided robust pooled estimates [16]. This evidence is highly relevant as it confirms that PA interventions can improve activity outcomes in LMIC settings. However, the review addressed PA promotion interventions more broadly and did not specifically focus on CBIs as a distinct implementation approach, nor did it examine in detail the implementation characteristics, such as delivery formats, fidelity, or adherence. Additionally, the review offers limited insights critical to embedding CBIs within existing community and primary health-care systems. Accordingly, an important unanswered question is which types of CBIs are structurally compatible with routine implementation within LMIC systems, rather than only effective under study conditions. Evidence from HICs also cannot be directly transferred to LMICs, because the two contexts differ markedly in terms of resources, infrastructure, implementation capacity, and stakeholder engagement in health systems with varied economies [17]. These differences are particularly relevant to CBIs, whose effectiveness often depends not only on intervention content but also on how the intervention is delivered—such as who implements it (e.g., trained community health workers, peer leaders, or educators), the level of training and supervision available, the intensity and frequency of contact, and the systems in place to monitor adherence and delivery quality. Such implementation features are often more resource-sensitive and context-dependent than the intervention message itself. Therefore, studying interventions specifically within LMICs is essential to generate contextually relevant evidence and to better understand the local implementation conditions needed for effectiveness [18–22]. The growing burden of physical inactivity and rising NCDs among adults in LMICs underscores the need to identify CBIs that improve PA and/or decrease SB. Synthesizing evidence on the effectiveness and implementation characteristics of CBIs will aid immediate policy-relevant actions [23–26]. This systematic review, therefore, aims to identify and synthesize evidence on the effectiveness of CBIs that promote PA and/or reduce SB among community-dwelling adults residing in LMICs, while also describing intervention delivery formats, behavioral scope, implementation features, and fidelity-related reporting.

## Methods

The protocol for this systematic review was registered with the International Prospective Register of Systematic Reviews (PROSPERO, CRD42024579461) [27]. The review follows the Preferred Reporting Items for Systematic Review and Meta-Analysis Protocols (PRISMA-P) 2015 guidelines.

### Search Strategy

The PICO (Patients/Population/Participants, Intervention, Comparison, Outcome) framework guided the search. The Population (P) included ambulatory adults aged > 18 years residing in LMICs, excluding those residing in HICs. LMICs were defined according to the World Bank income classification; this restriction was applied to generate context-specific evidence for implementation in resource-constrained settings [28]. The Intervention (I) comprised CBI, designed to promote PA and/or reduce SB. The Comparator (C) received routine care services, enhanced care, or no intervention. “Routine care” refers to usual information/services ordinarily available in the study setting, unrelated to a structured PA/SB program (e.g., standard clinic advice or generic health leaflets). “Enhanced care” denoted minimal, standardized add-ons to routine care (e.g., a single brief counseling/education session or generic printed materials), except for the ongoing study intervention. The Outcomes (O) focused primarily on changes in PA or SB, measured in terms of Metabolic Equivalents-min/week, PA intensity, and steps per day. PA and SB were measured using validated subjective (e.g., questionnaires and diaries) or objective (e.g., pedometers, smartphones, or accelerometers) assessments [29]. Secondary outcomes included alterations in anthropometric (e.g., Body Mass Index) and biochemical parameters (e.g., fasting or random blood glucose levels).

### Electronic Searches

Due to the diversity of interventions and health topics covered, the large population size, study types, and their outcomes, a multistage search strategy was developed to identify relevant publications. We searched for research studies published in PubMed, Embase (including *Medline and the Cochrane Library database*), and Scopus between January 1, 2000, and Dec 31, 2024.

Specific terms for the search strategy were piloted as database-controlled vocabulary in the databases searched. The searches included all possible variations of different terms (Supplementary material 1). In addition, reference lists of articles identified through database searches were examined to identify further relevant studies. Bibliographies of systematic and nonsystematic review articles were also examined to identify relevant studies. Cochrane reviews and systematic reviews in the Database of Abstracts of Reviews of Effects (DARE), retrieved from a wide range of OVID databases, were also explored to ensure that no relevant studies were missed. Only English-language articles were included in the review, because the reviewers responsible for screening, data extraction, and methodological quality assessment had working proficiency in English, and validated translation resources were not available. Search results were imported into bibliographic citation management software (Mendeley) to aggregate relevant review articles and exclude duplicate references [17]. Grey literature sources (Google Scholar, OpenGrey, ProQuest Dissertations & Theses, and public health evidence portals such as Health Evidence and PROSPERO) were also searched to locate additional eligible primary studies that might not have been indexed in the main databases. Because these platforms differ in their indexing structures and do not uniformly support complex, database-specific Boolean syntax, a simplified search approach was used, with keyword combinations adapted from the PubMed search strategy. Items such as annual or technical reports, dissertations, books, and conference papers were excluded from the final synthesis and were only reviewed for reference tracing.

### Inclusion and Exclusion Criteria

We included CBI studies that focused on improving PA and/or reducing SB among community-dwelling adults, or studies that depicted the effects of national or subnational health programs targeting PA or SB, using different study designs. Community-based interventions were defined as interventions implemented in community settings, such as communities or neighborhoods, workplaces, educational institutions, and transit areas. We did not include interventions implemented within or from hospital settings. The interventions aim to enhance individual behaviors or strengthen community capacity through approaches that view the community as a *target*, a *resource*, or an *agent* of change [30]. For consistency, studies were categorized as randomized controlled trials (RCTs) and quasi-experimental studies (e.g., pre–post and controlled before–and–after designs), including natural experiments. Studies were excluded at this stage if they lacked sufficient methodological information to permit quality assessment (using the Effective Public Health Practice Project (EPHPP) quality assessment tool) [31], such as an adequate description of the intervention design, evaluation framework, or outcome measures.

### Study Selection and Screening

After removal of duplicate records in Mendeley, titles and abstracts were screened against the predefined eligibility criteria. Screening was conducted in two sequential stages: first, title and abstract screening, followed by full-text screening of potentially eligible articles. Screening decisions were managed in Microsoft Excel using a structured screening sheet based on the predefined eligibility criteria. Given the large number of records retrieved, title and abstract screening were operationalized by dividing records between two reviewers (MV and GJ), who screened their allocated records against the PICO-based eligibility criteria. Records that were clearly ineligible were excluded at this stage, while records considered potentially eligible, unclear, or requiring further assessment were retained for full-text review. Reviewers were not blinded to author names, journal details, study institutions, or publication details.

Full texts of potentially eligible articles were then retrieved and assessed independently by both reviewers (MV and GJ) using the predefined inclusion and exclusion criteria. For each full-text article, both reviewers first recorded their independent eligibility decision and, where applicable, the primary reason for exclusion. Disagreements were discussed criterion-by-criterion until consensus was reached. A final inclusion or exclusion decision was made only after agreement between both reviewers. Reasons for exclusion at the full-text stage were documented and summarized in the PRISMA flow diagram. Inter-rater reliability for the full-text screening stage was assessed before consensus using Cohen’s kappa, based on the reviewers’ initial independent binary decisions on whether each full-text article should be included or excluded. The final set of included studies reflects the post-consensus decisions.

### Data Extraction

Data were independently extracted using an electronic data abstraction form adapted from the Cochrane Public Health Group Data Extraction and Assessment template (Supplementary material 2). It collected information on all necessary aspects in accordance with the Methodological Expectations of Cochrane Intervention Reviews (MECIR) standards [32]. The data abstraction form was piloted on a random sample of five included articles and modified as required based on team feedback. Full data abstraction began only after sufficient agreement (i.e., a percentage agreement > 90%). Each included study was abstracted by one team member (MV) and verified by a second reviewer (GJ). Data extraction for review studies included the aim, study characteristics, terminology used to describe the intervention, settings, intervention implementation duration, and follow-up durations. The studies were further categorized by intervention focus (e.g., PA promotion only, SB reduction, or broader lifestyle/risk-factor modification) and delivery formats (e.g., digital, peer group, community health-workers driven, or legislative interventions). Data were extracted for primary and secondary outcomes and methodological quality. In addition to outcome data, we extracted detailed information on implementation quality and fidelity parameters, such as who delivered the intervention, the Number of providers, provider training, Intervention quality assessment, and Fidelity/integrity assessment. Fidelity was conceptualized as the degree to which an intervention was delivered as intended. Additionally, we extracted information on any reported metrics related to adherence, attendance, engagement, or delivery consistency.

### Study Outcomes

The primary outcomes included physical activity and sedentary behavior. For this review, physical activity (PA) was conceptualized as any bodily movement produced by skeletal muscles that results in energy expenditure, including leisure-time, transport-related, occupational, and household activity. Sedentary behavior (SB) was conceptualized separately as waking behavior characterized by low energy expenditure while sitting, reclining, or lying. Therefore, SB was not treated simply as the absence of PA, but as a distinct behavioral construct.

PA was extracted exactly as reported in each study. Metrics included steps/day or steps/week (pedometer/device-based studies), MET-min/week (total or domain-specific), intensity-specific PA (moderate, vigorous, MVPA min/day or min/week), % Active (e.g., ≥ 600 MET-min/week using IPAQ/GPAQ thresholds; ≥30 min/day), total PA (min/week) or PA subscale scores from validated lifestyle questionnaires, and Walking prevalence or Leisure Time Physical Activity (LTPA)/transport PA proportions. Where studies reported domain-specific PA, we extracted the domain separately, including leisure-time, transport-related, occupational/work-related, and household PA. Where only total PA was reported, the outcome was retained as overall PA. All metrics were recorded as reported, capturing both continuous values (e.g., MET-min/week, minutes/week) and categorical classifications (% meeting PA guidelines). SB outcomes were extracted separately as sedentary time (minutes or hours/day), sitting time, screen time, or domain-specific sedentary behavior, as reported by the included studies. More importantly, Physical inactivity and SB were treated as distinct constructs: physical inactivity referred to not meeting recommended PA thresholds, whereas SB referred to low-energy activities performed while awake, usually in a sitting, reclining, or lying posture. Thus, an individual may meet PA guidelines while still accumulating high sedentary time.

Secondary outcomes included anthropometry (Body mass index (BMI), waist circumference, hip circumference/waist–hip ratio, and body fat percentage (BF%)) and clinical measures (systolic and diastolic blood pressure and fasting plasma glucose levels).

### Data analysis

Data extraction and analysis proceeded sequentially. First, we extracted and summarised the core study characteristics and assessed the methodological quality using the EPHPP quality assessment tool, as it is suitable for quantitative public health interventions and can be applied across diverse study designs, which was appropriate given the heterogeneity of included studies [31]. Each study was evaluated across six methodological domains (selection bias, study design, confounders, blinding, data collection methods, and withdrawals or drop-outs) on a three-point scale and was then classified as strong, moderate, or weak. Subsequently, the overall study quality was rated as 1 (strong) if no domain was rated weak, 2 (moderate) if one domain was rated weak, and 3 (weak) if two or more domains were rated weak. All ratings were performed independently by two reviewers (MV and GJ), with disagreements resolved by consensus [33–35]. Following this, we extracted detailed intervention characteristics for each study, including the primary purpose, theoretical basis, masking, intervention assignment, specific content delivered, delivery formats, additional components, and frequency/duration of implementation.

Interventions were then grouped into broad delivery formats based on shared structural and implementation features, which provided the basis for summarising intervention-specific effects. The results were summarized using descriptive statistics based on the direction of effect and statistical significance. Because the included studies exhibited considerable heterogeneity in intervention content, delivery mechanisms, outcome definitions, and analytical methods, meta-analysis was not feasible. We therefore conducted a structured narrative synthesis following SWiM (Synthesis Without Meta-analysis) guidance [36]. For each study, PA outcomes were reported as improved, unchanged, or worsened, along with their statistical significance. Secondary outcomes, including body mass index (BMI), waist circumference, hip circumference/waist–hip ratio, and BF%, as well as systolic and diastolic blood pressure and fasting glucose, were summarized using descriptive statistics.

Due to heterogeneity in interventions, outcomes, and study designs, we were unable to conduct a formal subgroup analysis. Instead, we used descriptive heat maps to summarize studies by outcome category across four predefined subgroup dimensions: intervention format, study setting, intervention complexity, and intervention duration. Interventions were classified by behavioral scope as PA/SB-focused when physical activity or sedentary behavior was the primary behavioral target, and as broader lifestyle or multi-behavior interventions when PA/SB was addressed alongside other lifestyle, educational, counseling, or cardiometabolic risk-factor components. These subgroups were informed by prior reviews of community-based PA interventions and by the implementation domains highlighted in WHO’s Global Action Plan on Physical Activity (GAPPA) [16, 37–40]. They were selected to examine how design, delivery context, program complexity, and duration may influence PA outcomes in real-world settings. In addition, heat maps were used to examine the percentage of studies in each subgroup reporting statistically significant PA improvements, to tentatively assess whether certain intervention types appeared more effective than others. Given the small number of studies within several subgroups, this assessment was explicitly exploratory. Therefore, subgroup-level summaries were restricted to categories with at least 5 studies, and subgroups in which at least 70% of studies reported significant effects were considered potentially promising. Each heat-map cell represented the number of studies within a given combination of subgroup and outcome category, depicted on a continuous color gradient (light yellow to dark blue) to indicate increasing frequency. Intervention duration was strictly defined as the *active implementation period* and categorized as short-term (≤ 6 months), medium-term (6–12 months), or long-term (> 12 months or ongoing initiatives**)**. This pragmatic categorization was specified a priori for descriptive synthesis and was informed by prior reviews [16, 41]. For the purposes of narrative synthesis and descriptive subgroup summaries, PA outcomes within each intervention subtype were classified into three categories: ‘Significant effect’, defined as a statistically significant improvement in at least one physical activity outcome compared with baseline or control (*p* < 0.05); ‘No effect’, defined as no statistically significant change in physical activity outcomes (*p* ≥ 0.05); and ‘Mixed/partial effects’, defined as improvements in some physical activity indicators but not others, or effects observed only in specific domains, subgroups, or time points.

## Results

### Search Results

**Fig. 1:**
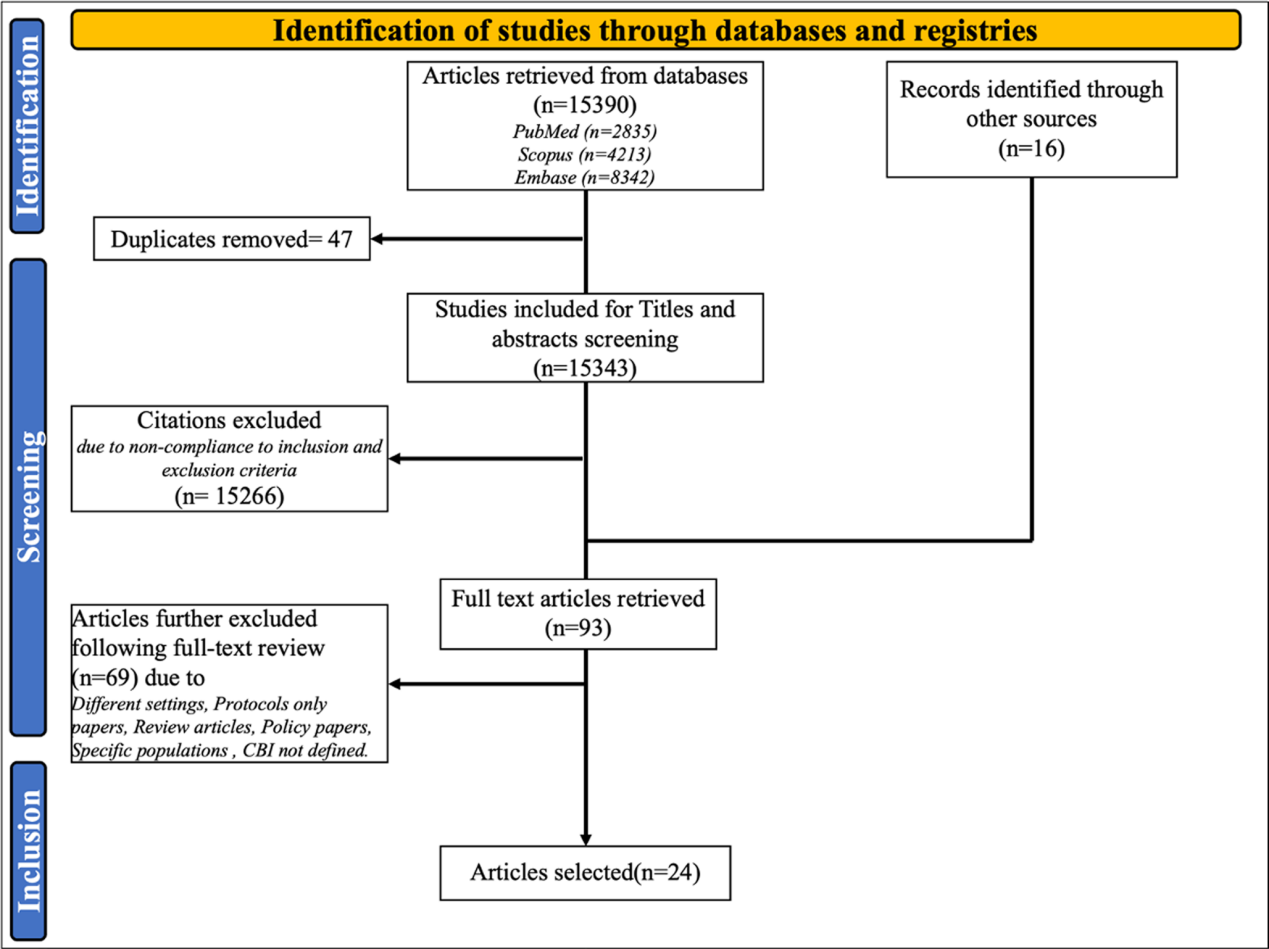
PRISMA flow chart

A total of 15,390 records were identified from database searches (PubMed = 2,835; Scopus = 4,213; Embase = 8,342). After removing 47 duplicates, 15,343 records were screened by title and abstract, with 15,266 excluded for not meeting the inclusion criteria according to the PICO format. Common reasons for exclusion included studies conducted in non-relevant settings, populations outside the target group, interventions that did not meet the definition of CBI (e.g., conducted in hospital settings, paid gyms, or rehabilitation centers), or publications such as reviews, protocols, and policy papers. An additional 16 literature records were identified from other sources through citation tracking and snowballing. Grey literature sources were also searched; however, no eligible grey literature document was identified or included in the final synthesis. Ninety-three full-text articles were assessed for eligibility by both reviewers. Inter-rater reliability for the initial full-text inclusion/exclusion decisions was high (Cohen’s κ = 0.83). After consensus, 69 full-text articles were excluded for various reasons (interventions delivered in settings not meeting inclusion criteria (*n* = 20), protocol-only papers (*n* = 1), review articles (*n* = 15), policy papers (*n* = 4), studies on specific populations not aligned with inclusion criteria (*n* = 11), and interventions not clearly defined as CBIs (*n* = 18). We finally selected 24 studies for inclusion in the review. The detailed screening and selection process is shown using the PRISMA (Preferred Reporting Items for Systematic Review and Meta-Analyses) flow diagram (Fig. 1).

### Study Characteristics, Methodological Quality, and Fidelity Reporting

Of the 24 included studies, 11 were randomized controlled trials (RCTs) [42–52], and 13 were quasi-experimental or natural experiments (Table 1) [53–65]. Studies were conducted across nine countries, most frequently Iran (*n* = 7) [42, 45, 49, 52, 54, 58, 63], followed by China (*n* = 4) [43, 44, 47, 55], India (*n* = 3) [59–61] and Brazil (*n* = 2) [48, 65], Colombia [56, 64], and Malaysia [50, 57]. Other countries included Jordan, Pakistan, and Nigeria [46, 51, 62]. Sample sizes ranged from 46 to > 12,000 participants, with 13 studies enrolling > 300 individuals. The mean age of participants ranged from 18 to 69.4 years. Seven studies recruited only women, while others included students, employees, or adults with NCD risk factors. Female representation varied from 40.6% to 100%. Funding was reported in 16 studies, while 5 did not disclose funding sources. Interventions were delivered in community/health center settings (*n* = 14), educational institutions (*n* = 5), workplaces (*n* = 3), and multi-setting/community-wide programs (*n* = 2). Based on the EPHPP tool, most studies were rated weak (*n* = 17), five were rated moderate, and only two were rated strong. Supplementary material 3a provides additional details on who delivered the intervention, the number of providers involved, and provider training.

**Table 1:** Basic characteristics of the studies included in the review (*n* = 24)

Study Id	Geographic location	Study setting	Inclusion criterion	Age	Funding	Intervention quality assessment	Fidelity/integrity assessment	Quality assessment rating
Study Design: Randomised control trials								
Pazoki R, 2007 [42]	Iran	Community/ Health Center	25–65 year women who had not history or evidence of angina pectoris, myocardial infraction, cerebral stroke, renal disease, severe arthritis, lung disease or drug consumption	25–65	Yes	Surveys and home visit logs assessed intervention adherence and impact on physical activity behaviors	Volunteer logs and session delivery check; descriptive only	Moderate
Chao, 2012 [43]	China	Community/ Health Center	participants were aged 60 and over; and local permanent resident	≥ 60	Yes	Health evaluations, biometric assessments, and self-reported adherence tracking were conducted at regular intervals	Attendance and session-delivery records; descriptive	Moderate
Gholamnia SZ, 2017 [45]	Iran	Community/ Health Center	being literate, wishing to participate, not presently participating in other research, and no history of chronic conditions, mental and/or disabling disorders	18–64	NR	Quality checks included digital tracking of user engagement and periodic feedback from participants	Engagement via software analytics; no numerical values	Weak
Memon, 2018 [46]	Pakistan	Educational institutions	undergraduate medical students who were overweight or obese, measured by the Asian body mass index (BMI) and active Android/Iphone/Windows smartphone users	18–25	Partial	Weekly step count reports, participant self-assessments, and periodic researcher evaluations were used for quality monitoring	Step logs and self-assessments; fidelity not quantified	Weak
Mui et al., 2018 [47]	China	Community/ Health Center	Physically inactive participants with atleast 1 first-degree relative with physician-diagnosed diabetes;	≥ 30	Yes	System-based standardization; call frequency and duration predefined	Standardized automated delivery ensured consistent intervention fidelity; but no formal fidelity assessment reported	Weak
Meurer, 2019 [48]	Brazil	Community/ Health Center	Frequent (regular attendance according to the HAP sheets) users of the HAP,	> 20	Yes	Regular monitoring of participant engagement, self-reported adherence, and session attendance tracking	Attendance and session-delivery monitoring; non-numeric	Weak
Mouodi S, 2019 [49]	Iran	Community/ Health Center	living in Amirkola; able to read and write and not the medical history of diabetes or hyperlipidemia	40–60	Yes	Periodic evaluations through participant surveys, adherence tracking, and performance assessments of educators	Facilitator checklists and attendance; no percentages	Weak
Hidrus A, 2020 [50]	Malaysia	Educational institutions	Ambulartory adults living with Type 2 diabetes, able to read/write Malay, no comorbidities that hinder PA	≥ 18	Yes	Assessment through participant adherence tracking, questionnaire evaluations, and physical activity logs	Activity logs and researcher monitoring; descriptive only	Weak
Alsaleh E, 2023 [51]	Jordan	Educational institutions	University students	18–25	NR	Regular participant feedback, adherence tracking and assessment of progress towards physical activity goals	Attendance and participant logs; no numeric fidelity	Weak
Ansari K, 2023 [52]	Iran	Community/ Health Center	Overweight or obese women	40–60	Yes	Digital tracking of engagement with mobile interventions and periodic assessment of behavioral changes	Digital engagement tracked; no quantified adherence	Weak
Li et al., 2023 [44]	China	Community/ Health Center	Community-dwelling adults if they were able to answer telephone calls, walk 400 min 15 min, walk without any assistance, and complete the Timed Up & Go test	≥ 60	Yes	NR	NR	Strong
Study Design: Quasi experimental								
Rabiei K, 2010 [63]	Iran	Community/Health Center	whole population of the urban and rural areas of 2 study areas was included. Intervention community divided into 8 target groups: women, workers, children, young people, cardiovascular patients and their family members, high risk population, health professionals and NGO members	Not defined	NR	NR	NR	Weak
Torres A, 2013 [64]	Colombia	Multiple	Participants of Ciclovia and Cicloruta programs	≥ 18	Yes	Direct observation of infrastructure use and surveys on changes in physical activity patterns	Natural experiment—fidelity not applicable	Weak
Lv J, 2014 [55]	China	Multiple (neighbourhoods, schools, workplaces & community health centres)	Community dwelling adults residing in two intervention and one comparsion arm of selected communities	18–64	Yes	NR	NR	Weak
Anthony, 2015 [53]	China, India and Mexico	Multiple (Health centers, workplaces, schools, and the community)	Community based adults 18–64 years (from health centers, workplaces, schools, and the community)	18–64	Yes	Workplace health assessments and structured employee feedback surveys	Workplace supervisor records; adherence not quantified	Weak
Baghianimoghaddam et al., 2016 [54]	Iran	Workplace	Female employees, sedentary jobs, no medical conditions preventing PA participation	20–50	Nil	Step count data analysis, self-reported activity logs, and qualitative feedback from participants	Session-delivery and activity monitoring; non-numeric	Moderate
Simões EJ, 2017 [65]	Brazil	Community/Health Center	Residents of participating cities in Pernambuco, Brazil	≥ 16	Yes	Regular monitoring of class attendance, participant engagement, and impact evaluations	Employer logs and facilitator records; not quantified	Moderate
Peyman, 2018 [58]	Iran	Community/Health Center	having a mobile phone and the ability to use it, access to the internet, ability to use a computer and the internet, and willingness to participate	> 18	Nil	Platform analytics, engagement metrics, and participant feedback surveys measured intervention effectiveness	Platform-use analytics and feedback; no percentages	Weak
Pentakota et al., 2019 [59]	India	Educational institutions	Undergraduate medical students with smartphones, not involved in other weight loss programs	18–24	Nil	User engagement analytics, app usage metrics, and participant feedback surveys	Self-monitoring logs and feedback; no numeric fidelity	Weak
Mathew V, 2019 [60]	India	Workplace	Software company employees working in managerial position or as team heads not using pedometers	≥ 30	Yes	Workplace health assessments and structured employee feedback surveys	Attendance and completion logs; descriptive only	Weak
Mathews E, 2021 [61]	India	Community/Health Center	Sedentary women but not with the physical deformities like bedridden, pregnant, lactating and disesase conditions	15–64	Yes	Evaluation through structured feedback from participants and ongoing monitoring of attendance at group sessions	Attendance records and structured feedback; descriptive	Weak
Eze II, 2021 [62]	Nigeria	Workplace	All adult registered members of the market traders receiving care through primary health facilities	Adults- range NR.	NR	NR	Adherence encouraged using activity logbooks (diaries), weekly SMS reminders, monthly reinforcement sessions, and attendance via outreach health post registers; No formal fidelity metrics or quantitative adherence measures	Strong
Baldovino-CL, 2023 [56]	Colombia	Community/Health Center	Adults (aged ≥ 18 yrs) living in intervention or control areas for at least 2 years and not planning to move within the next 2 years	≥ 18	Yes	Monthly reports on attendance, participant engagement, and fitness progress tracking	Natural experiment—fidelity not applicable	Weak
Cheah et al., 2024 [57]	Malaysia	Multiple (workplace, educational)	Employees from eligible campus workplaces (3-story buildings, ≥ 4 staircases, predominantly white-collar staff)	20–60	NR	Environmental quality assessed using the Environmental Assessment Tool (EAT)	Not reported	Moerate

Fidelity reporting varied substantially across studies, and only a subset specified quantitative adherence thresholds or benchmark criteria. As shown in Table 1, fourteen studies reported some form of structured monitoring, including attendance logs, digital engagement metrics, adherence tracking, session-completion records, or facilitator checklists. In several studies, engagement or adherence was described using app-use metrics, attendance records, or facilitator-reported completion. Details of intervention delivery personnel, number of providers, and provider training are presented in Supplementary material 1. Digital interventions typically required fewer direct providers, used automated platforms, app interfaces, or messaging systems; accordingly, training requirements were minimal or limited to orientation, and fidelity was inferred through digital engagement metrics rather than direct observation [46, 58, 59]. In contrast, peer/CHW-led interventions involved trained health workers, facilitators, or peer leaders, often with structured training sessions and supervision; however, fidelity reporting varied from descriptive attendance logs to limited documentation of adherence thresholds [42, 43, 62]. Environmental or multi-setting interventions depended on multiple stakeholders and institutional actors, and fidelity was infrequently quantified beyond participation counts or exposure descriptions [56, 57, 64]. The two strongly rated studies reported clearer implementation structures within community or workplace systems [44, 62].

### Intervention Design, Content, and Delivery Formats

Table 2 summarizes the intervention purpose, theoretical basis, assignment approach, content delivered, main delivery format(s), additional formats, and implementation duration. In most studies (*n* = 15), PA promotion was delivered as a part of broader lifestyle/multi-behavior interventions, whereas 9 studies focused primarily on PA. Furthermore, 20 studies (83.3%) used a conceptual/theoretical framework to design their interventions. The most frequently cited approaches were general behavioral change or health promotion–oriented frameworks, reported in seven studies [43, 49, 52, 54, 58, 60, 64], followed by Social Cognitive Theory (*n* = 3; [48, 51, 61]), socioecological or multilevel models (*n* = 3; [44, 53, 55]), the theory of planned behavior (*n* = 2; [45, 61]), self-determination and motivation-based models (*n* = 2; [47, 50]), and behavioral economics or incentive-based frameworks (*n* = 2; [46, 59]), community-based participatory research approach (*n* = 1; [42]), and a necessity-versus-choice framework (*n* = 1; [56]).

**Table 2:** Details of the delivered interventions in the studies included in the review segregated as per the primary intervention type

Study Id	Primary Purpose of the intervention	Theoretical basis of the intervention	Masking	Intervention assignment	Content of the intervention given to the intervention group	Main Format(s) used to deliver the intervention	Additional formats	Frequency and duration of intervention.
Digital/Technology-Assisted								
Baghianimoghaddam et al., 2016 [54]	Assess the effectiveness of pedometers in motivating to walk more.	Team-based motivational theories	Not applicable.	Pre-post	Team-based activities with pedometer tracking and regular feedback on activity levels.	Team-based activities coordinated by team leaders.	Weekly step-count logs and activity feedback.	4 months
Gholamnia SZ, 2017 [45]	Promote physical activity among postpartum women and enhance behavior.	Theory of Planned Behavior, coping planning	Yes (Double-blind)	Parallel Assignment	Multimedia-based software, self-paced lessons through videos shared via WhatsApp	Digital video-based intervention shared via WhatsApp	adherence and engagement logbooks	daily 10-minute video-based physical activity for 4 months
Peyman, 2018 [58]	Assess the impact of digital intervention in promoting physical activity	health intervention for behavioral change	Yes (Single-blind)	Quasi-experimental study with intervention and control groups.	Digital media-based intervention for educating and motivating women to engage in physical activity.	Digital platforms, including mobile phone applications, online educational content, and SMS reminders.	Multimedia educational CDs, online self-assessment tools, and interactive chat rooms for motivation.	Interventions delivered continuously over 2 months.
Mui et al., 2018 [47]	To evaluate the efficacy of a culturally adapted telephone linked care platform to increase physical activity among physically inactive adults at high risk of developing diabetes	Established behavioral change theories.” (Stages of Change, self-efficacy, goal-setting embedded in TLC logic)	Non blinded	Parallel assignment	Automated, individually tailored telephone counseling including weekly self-report of PA, personalized feedback based on behavioral profile (stage of change, self-efficacy, prior responses), interactive goal negotiation and goal setting, and motivational messaging to promote moderate-intensity PA	Fully automated telephone-based counseling system	**-**	Weekly automated telephone counseling calls (5–10 min) for 6-months.
Memon, 2018 [46]	Assess the impact on physical activity and weight loss.	Incentive-based intervention behavior modification.	No	Parallel Assignment	Weekly financial incentives for reaching specific step count goals tracked via a smartphone app.	Smartphone-based.	Financial incentives, step tracking via app.	Weekly intervention for 5 weeks
Pentakota et al., 2019 [59]	Examine smartphone app efficacy in promoting physical activity.	Behavioral economics and technology-enabled intervention.	Not applicable.	Pre-post	Installation of a mobile app to monitor and provide feedback on physical activity levels.	Smartphone app monitoring and reminders.	Mobile notifications and motivational features.	1 month
Mathew V, 2019 [60]	Evaluate the effect of pedometer use on increasing physical activity levels.	Health behavior change models emphasizing feedback and self-monitoring	Not applicable.	Pre-post	Distribution of pedometers, goal setting, and monitoring activity levels among employees.	Direct instruction on pedometer usage and feedback sessions.	Motivational interviews and follow-ups.	daily for 7 days
Hidrus A, 2020 [50]	To improve motivation and amount of physical activity.	Self-determination theory and achievement goal theory	Yes (Double-blind)	Parallel Assignment	Brain Breaks^®^ via WhatsApp; participants followed daily; adherence monitored via logbooks	Digital delivery via WhatsApp group	motivational video, adherence logbook, reminder messages	Daily PA videos (10 min/day), video, changed weekly for 4 months
Alsaleh E, 2023 [51]	Improve health behavior (Broader lifestyle / multi-behavior)	Social Cognitive Theory	No	Parallel Assignment	individualized consultations for behavioral change	text messages, calls reminders and interactions with a facebook Page	-	Monthly for 6 months
Ansari K, 2023 [52]	Compare effects on physical activity and anthropometric indices.	Empowerment using digital health tools, leveraging Social Influence and Behavioral Change Theory	No	Parallel Assignment	text messaging and mobile social networking to provide information on the importance of physical activity, overcoming barriers, and self-monitoring techniques.	Mobile-based digital intervention through text messaging and social networking apps.	Self-monitoring logs, instructional videos, and interactive group chats.	three times/week for 3 months.
Peer-Led / CHW–Facilitated								
Pazoki R, 2007 [42]	To assess the effectiveness of a community-based lifestyle modification program in increasing physical activity. (Broader lifestyle / multi-behavior)	Community-Based Participatory Research approach	No blinding	Parallel Assignment.	lifestyle modification program based on the Choose to Move program with weekly home visits by trained volunteers who provided audio-taped activity instructions, music, and educational materials.	Face-to-face home visits by trained community health volunteers, who provided education and guided participants in physical activity routines.	Printed educational booklets, audio-taped exercise instructions, and a structured volunteer support network.	weekly one-on-one educational visits for 2 months, conducted by trained community volunteers.
Chao, 2012 [43]	Evaluate the impact of community-based health management on elderly health. (Broader lifestyle / multi-behavior)	behavioral and health education theories	Yes	Parallel Assignment.	Monthly health evaluations, tailored exercise programs, diet advice, psychological support, and education on health management.	Direct community health services and monthly follow-ups by trained staff.	Telephone consultations, health lectures, and distribution of health materials.	=once per month for 1. years.
Anthony, 2015 [53]	Reduce health risk factors, including physical inactivity, in workplaces (Broader lifestyle / multi-behavior)	Socio-ecological model	Not applicable.	Parallel Assignment.	Culturally tailored interventions addressing tobacco use, diet, and physical activity.	Workplace-based health promotion programs.	Educational materials and environmental changes.	18–24 months
Mouodi S, 2019 [49]	Evaluate the effect of lifestyle modification interventions on nutritional behaviors, physical activity, anthropometric measurements, fasting blood sugar, and serum lipid profile (Broader lifestyle / multi-behavior)	principles of health promotion	No	Parallel Assignment	High-intensive intervention: Weekly group nutrition and physical activity training classes, individual nutrition consultation, educational package, and weekly aerobic exercises. Low-intensive intervention: Weekly group nutrition training classes, individual nutrition consultation, and educational package.	Face-to-face group discussions and individual consultations.	Educational package including a book and a DVD	High-intensive intervention: Weekly for 4 months. Low-intensive intervention: Weekly for 4 months.
Meurer, 2019 [48]	to motivate people to adopt a healthy lifestyle (Broader lifestyle / multi-behavior)	Social cognitive theory	No	Parallel Assignment	VAMOS strategy based educational intervention. Self-monitoring, self-incentive and guided action to reach predetermined goals, identification of social support, identification of individual barriers, outcome expectancies, facing changes in PA, and dietary habits.	Group discussions, practical workshops, self-monitoring tools, and goal-setting exercises.	-	weekly group sessions for 3 months.
Mathews E, 2021 [61]	To address the gap in knowledge, increase selfawareness, enhance social support, and engage in sustainable behaviour change. (Broader lifestyle / multi-behavior)	Behavioural theories of reasoned action and planned behaviour, social cognitive theory	Yes (Single-blind)	Parallel Assignment	NCD Risk assessment, Educational workshop, Group counselling, Goal setting and review, Peer support.	Peer-led support groups, community mobilization, and structured workshops.	Participant handbook, peer leader training, and printed educational materials.	3 phases: Intense phase (0–3 months), less intense phase (4–6 months), no intervention (7–12 months)
Eze II, 2021 [62]	To determine the effect of on-site behavioural modification intervention on lifestyle risk factors of hypertension (including PA) among adult market traders.	NR	Yes (Single-blind)	Quasi-eperimental	Lifestyle counselling including PA advice, exercise demonstrations, and behavioural reinforcement; participants received guidance to engage in regular PA (≥ 30 min/day), supported by follow-up reminders.	Face-to-face, on-site group education and counselling delivered in market settings by health professionals	Weekly SMS reminders, monthly reinforcement sessions, use of logbooks/diaries	Daily self-monitoring using activity logbooks; weekly bulk SMS reminders; monthly health awareness campaigns.
Li et al., 2023 [44]	To evaluate the effects of a multilevel intervention for increasing leisure-time activity levels in Chinese older adults.	Socioecological model (multilevel intervention at individual, interpersonal, and community levels)	Yes (Single-blind)	Cluster randomized allocation	Multilevel intervention comprising individual-, interpersonal-, and community-level components like social capital, and environemtnal factors.	Face-to-face and Telephone-based counselling, training sessions, printed material, peer group, coaching, and group sharing	**-**	8-week active multilevel intervention, followed by outcome assessments at 4 weeks, 8 weeks, 6 months, 12 months, and 24 months post-baseline (no active intervention during follow-up).
Environmental /Infrastructure Based								
Rabiei K, 2010 [63]	To assess the effect of a comprehensive CBI for increasing PA	NR	Not applicable	Quasi-eperimental	Multicomponent Population-wide PA promotion including educational campaigns, environmental restructuring to support active transport and recreation, and enforcement of school- and worksite-based physical activity policies.	Community-wide environmental and policy interventions implemented through municipal authorities, health systems, schools, and worksites.	Mass media campaigns, community events, workplace and school-based activities, and public educational materials.	Multiple ongoing, & recurrent intervention implemented over 2 years.
Torres A, 2013 [64]	To increase physical activity and improve health outcomes in urban environments.	Behavioral change theory	Not applicable.	Parallel Assignment.	Regularly closing streets for recreational use, creating safe environments for walking and cycling.	Direct community engagement through street closures.	Promotional events, community engagement activities.	weekly on Sundays and holidays
Lv J, 2014 [55]	To assess the short-term impact of a comprehensive, community-based multilevel intervention on knowledge, beliefs and practices with respect to smoking, physical activity and diet in Hangzhou, China	Socioecological model	No	Quasi-experimental	Multicomponent community-based physical activity promotion program including community mobilisation, structural change, health education and social marketing	Distributing messages through various community-based channels on a small scale, providing free screening and medical consultation, holding community-wide events on special days, implementing and enforcing smoking bans and restrictions and training health professionals.		A 2-year intervention programme was begun in mid-2009 in the intervention areas and continued until mid-2011.
Simões EJ, 2017 [65]	To evaluate the impact of a large-scale, community-based physical activity intervention (AC-P) on population-level leisure-time physical activity		No	Pre-post	Daily supervised PA classes in public open spaces, including parks and plazas, led by trained physical educators, and also dietary counseling, hypertension screening, and coordinated referrals.	Supervised group exercise classes in public spaces, combined with health education and community outreach.	Public awareness campaigns, local government-supported infrastructure and collaboration with the Ministry of Health.	daily PA sessions for participants, with continuous program expansion across different cities over 3 years.
Baldovino-CL, 2023 [56]	To assess the effect of a cable car and integrated urban improvements on physical activity	necessity vs. choice framework	No blinding	Quasi-experimental	Installation of the TransMiCable cable car system with supporting urban improvements	Not applicable (city-wide infrastructure project, not delivered by CHWs)		One-time infrastructure implementation; follow-up at 1 year
Cheah YX., 2024 [57]	To determine the outcomes of a multicomponent workplace environmental intervention incorporating physical activity self-regulation (PASR) to promote physical activity among employees	Not explicitly stated	No blinding	Cluster assigned Quasi-experimental	Multicomponent intervention including a 30-min face-to-face PASR briefing; goal setting and self-monitoring using pedometers; environmental prompts (posters, banners, stair point-of-decision prompts); organizational supports (PA education and stretching videos, written policies, virtual step challenges); and incentive-based reinforcement (certificates for achieving step goals).	Physical environment modification, organizational environmental changes, one time in-person briefing, and digital pedometers as delivery aids	Supplementary digita materials; pedometers’virtual step challenge.	Outcomes measured at 0, 3 and 6 months

### Study Outcomes

**Fig. 2:**
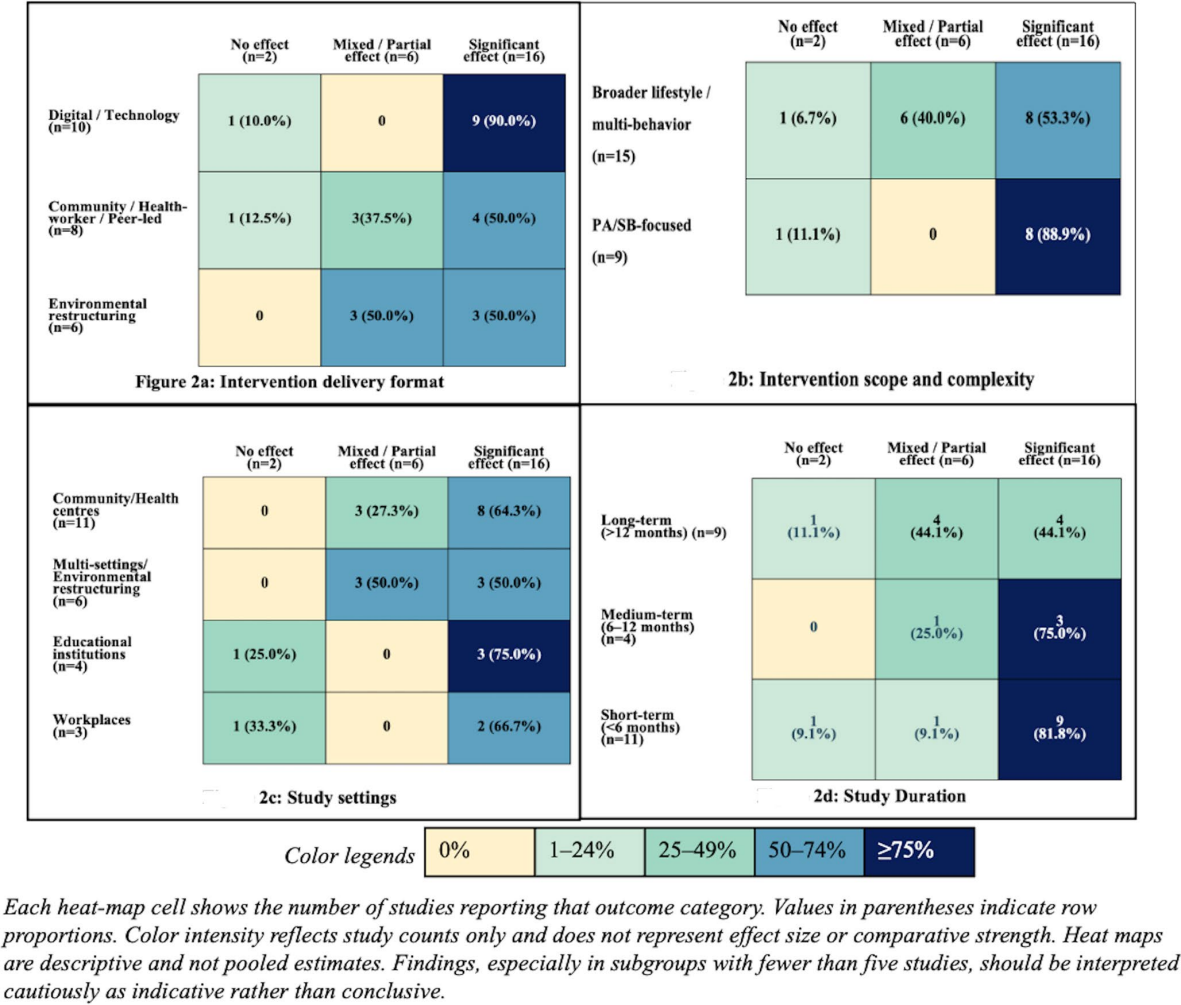
Summary of Physical activity outcomes* across (**a**) intervention type, (**b**) intervention complexity, (**c**) study setting, and (**d**) study duration in low- and middle-income countries (LMICs)

We segregated interventions into three main types: digital/technology-assisted interventions (*n* = 10), peer-led or CHW-facilitated programs (*n* = 8), and environmental/infrastructure-based approaches (*n* = 6). Delivery formats varied substantially across the three formats. Detailed format-specific primary outcome findings are presented in Tables 3, 4 and 5, while Fig. 2 provides exploratory heat-map summaries across the four predefined subgroup dimensions. Digital interventions (that deployed mobile apps, WhatsApp, pedometers, and SMS prompts) were mostly self-guided, with periodic prompts and support [45–47, 50–52, 54, 58–60]. Studies involving Peers/community health workers provided in-person, structured intervention through group-based peer sessions, CHW-led counseling visits, and peer-supported goal-setting workshops [42–44, 48, 49, 53, 61, 62]. However, Environmental/ infrastructure interventions primarily operated through exposure to policy or built-environment changes (such as open-street programs, upgraded parks/playgrounds, dedicated walking/cycling paths, transport-linked pedestrian corridors, and legislative changes), with limited direct interpersonal delivery compared with peer/CHW models [55–57, 63–65]. The comparator or the control groups in most studies (*n* = 18) typically received routine care, generic educational materials, or no structured intervention (data not tabulated).

### Primary Outcomes

Across the 24 included studies, PA was most commonly (*n* = 16) measured using MET-based measures (e.g., MET-minutes/week or MET-minutes/day). In most cases (*n* = 13), these MET values were derived using standardized self-reporting instruments such as the IPAQ or GPAQ. Categorization of participants according to IPAQ/GPAQ cut-offs (e.g., % meeting recommended PA levels) was reported in 13 studies. Intensity-specific outcomes such as MVPA were assessed in 8 studies, and domain-specific activity (e.g., leisure-time, work-related, or transport-related PA) was reported in 10 studies. Step counts were used in 5 studies, typically measured through pedometers (*n* = 4) or smartphone-based tracking (*n* = 1). Across measurement approaches, most studies (*n* = 16, 66.7%) reported statistically significant improvements in at least one PA outcome, 6 (25.0%) demonstrated mixed or partial effects, and 2 (8.3%) studies reported no significant effect. Only two studies assessed SB as an outcome, namely Meurer et al. (accelerometer-measured sedentary time) and Li et al. (self-reported leisure-time sitting) [44, 48]. Estimating the direct pooled estimates was not feasible, so we grouped the results by four pre-specified intervention characteristics, including intervention type, study settings, complexity, and duration of the intervention, and summarized the direction of change in PA within each stratum (Fig. 2).

#### • Intervention Delivery Format

- **Digital/Technology Assisted**: Nine of 10 studies reported significant improvements in PA (Table 3; Fig. 2a) [45–47, 50–52, 54, 58–60]. The improvements varied across studies, with several interventions showing statistically significant increases in MET-minutes per week or step counts. For example, individual studies reported increases of more than 2700 MET-min/week [58], over 3000 steps per day [60], and a rise in the proportion of active participants from 3% to 62% [45]. In one study using an incentive-based mobile app, an increase in PA was observed in the first 4–5 weeks, but this was not maintained at later follow-up [46].
- **Peer-led/CHW-Based Interventions**: Four of eight studies reported significant improvements (50%) [42, 43, 49, 62] and three demonstrated mixed/partial effects (37.5%) (Table 4) [44, 48, 61]. The magnitude and sustainability of improvements varied across studies. For instance, Pazoki et al. reported an increase of more than 100 MET-min/week (*p* < 0.05), and Mouodi et al. reported gains ranging from 900 to 1800 MET-min/week [42, 49]. Other studies showed mixed results over extended follow-up periods [61].
- **Environmental Restructuring**: Of the six studies (Table 5), three (50.0%) showed significant improvement, while three (50.0%) demonstrated mixed/partial effects. In the Ciclovía and city program studies, transport-related PA levels were higher among participants exposed to the program (odds ratio: 10.3; 95% CI: 7.9–13.5) [64, 65]. Baldovino 2023 reported an 11% increase in walking, with no statistical change in MVPA [56]. Rabiei et al. reported an increase in the intervention community’s LTPA, whereas transport-related PA showed no significant change; in contrast, both domains declined in the reference community [63]. Cheah et al. demonstrated improvements in step counts and selected PA domains, although between-group differences in total MET-min/week were not statistically significant [57].
- **Intervention Complexity**: No clear difference in effectiveness patterns was observed between PA/SB-focused and broader lifestyle interventions across delivery formats (χ² = 4.80, *p* = 0.09). Among PA/SB-focused interventions, 8 of 9 (88.9%) reported statistically significant improvements in PA(Fig. 2b). Likewise, 8 of 15 (53.3%) broader lifestyle interventions reported significant improvements in at least one PA outcome [43, 49].
- **Study Settings**: Among three interventions limited only to the workplace (Fig. 2c), two reported statistically significant improvements in PA [54, 60]. Likewise, three of four (75%) interventions delivered in educational institutions resulted in significant improvements in PA [50, 51, 59]. In community settings, 8 of 11 (64.3%) interventions reported significant improvements in PA [42, 43, 45, 47, 49, 52, 58, 62]. The multi-setting or community-wide studies also reported mixed/partial [56, 57, 63] and significant effects (50% each) [55, 64, 65].
- **Intervention Duration**: Among short-term interventions (≤ 6 months), 9 of 11 (81.8%) [42, 45, 49, 50, 52, 54, 58–60] reported statistically significant PA improvements (Fig. 2d). For medium-term interventions (6–12 months), 3 of 4 (75%) reported significant improvements [47, 51, 62]. In contrast, among longer-duration or ongoing interventions (*n* = 9), 4(44.1%) studies reported significant improvements [43, 55, 64, 65], while the other 4 (44.1%) reported mixed effects [44, 56, 61, 63].

**Table 3:** Summary of digital/technology-assisted interventions and their impact on physical activity in low- and middle-income countries (LMICs) included in the review (*n* = 10)

Study ID	Sample size (intervention/control)	Follow-up (months)	PA measure(s)	Measurement of PA	Intervention effect (baseline → endline)	Control effect (baseline → endline)	Reported comparative effect (intervention vs. control)	Significance	Conclusions
Baghianimoghaddam et al., 2016 [54]	77 / 77	4	Steps/day; Domain-specific MET-min/week	Pedometer +IPAQ	Workday steps: 4715 → 8279 (*p* = 0.001) Non-workday steps: 4339 → 6438 (*p* = 0.001) LTPA: 325 → 1086 MET-min/wk (*p* = 0.001)	Workday steps: 3806 → 4118 (NS) Non-workday steps: 3655 → 3529 (NS) LTPA: 321 → 369 (NS)	Intervention ↑ steps/day and leisure-time PA, but no effect on other PA domains (Significant ↑, domain-specific)o	LTAP *p* < 0.01; Other domains NS	Pedometer program ↑ daily steps and leisure-time PA; but no change in other domains
Gholamnia, 2017 [45]	65 / 65	3 & 6	MET-min/week; % Active (IPAQ); Intensity-specific PA	IPAQ	% Active: 3% → 62% (3 mo), 55% (6 mo) %MVPA: 8% → 62% (3 mo), 57% (6 mo)	% Active: 2% throughout %MVPA: 10% throughout	Large and significant ↑ in proportion of active women in intervention with waning over times vs. control (Significant ↑)	*p* < 0.001	WhatsApp video lessons led to marked improvement in women’s PA
Peyman, 2018 [58]	180 / 180	2	MET-min/week	IPAQ	992 → 3604	838 → 882	Intervention group showed about 2700 greater MET-min increase compared with control (Significant ↑)	*p* < 0.001	Digital education via mobile & online tools was highly effective
Memon, 2018 [46]	28 / 28	1.25	Steps/week	Mobile Phone based app.	Decline after week 5 (no sustained ↑)	Stable	No significant between-group difference (No effect)	NS	Incentive-only smartphone app failed to sustain PA gains
Mui et al., 2018 [47]	109/101	6	MVPA Duration (min/week); % meeting WHO PA recommendations	Self-reported IPAQ-SF (Chinese version)	Duration: ↑ by + 19.56 min/week per month % meeting WHO PA recommendations: 0% → 42.2%(3 months) → 44.0% (6 months)	Duration: ↑ by + 5.35 min/week per month % meeting WHO PA recommendations ↑ from 0% (baseline) →6.9% (3 months) → 13.9%(6 months)	Significantly higher likelihood of meeting WHO PA recommendations in the intervention group at 3 (Relative risk = 6.09; 95% CI 2.99–12.79) and 6 (3.18; 1.91–5.42) months compared with control.	*p* < 0.001	Automated telephone counseling significantly increased the likelihood of meeting WHO PA recommendations among physically inactive adults.
Pentakota et al., 2019 [59]	350 (pre-post)	1	Domain specific METS/min; Total PA METS; % Active (IPAQ)	GPAQ & Mobile Phone based app.	Work PA: 539 → 541 METS Travel PA: 223 → 223 Leisure-time PA: 0 → 1260 Total PA: 890 → 1870 %Active: 81% → 91%	No control	Pre–post intervention showed substantial ↑ in leisure-time and total PA, with more participants achieving adequate PA (Significant ↑)	*p* < 0.001	Smartphone monitoring app boosted PA, especially leisure-time and adequacy; work and travel PA unchanged
Mathew V, 2019 [60]	46 (pre-post)	0.25 (1 wk)	Steps/day	Pedometer	6963 → 9834 steps/day	No control	Short-term pre–post ↑ by ~ 3000 steps/day (no control) (Significant ↑)	*p* < 0.05	Short-term pedometer + peer-led program was effective
Hidrus A, 2020 [50]	37 / 33	4	Total PA (min/week)	IPAQ-M:	0.570 → 800 min/week, Approx (steady ↑)	390 →180 min/week approx (steady ↓)	Mean difference: +358 min/week (95% CI: 149–567) (Significant ↑)	*p* = 0.001	Brain Breaks videos via WhatsApp significantly ↑ PA over 3 months; control group showed marked decline
Alsaleh E, 2023 [51]	76 / 70	6	MET-min/week; Duration (min/wk); Steps/day; % Active (IPAQ)	IPAQ + Pedometer	MET-min: 462 → 985 Duration: 58.0 → 88.0 Steps/day: 6396 → 9396 % Active: 32% → 81%	MET-min: 460 → 466 Duration: 60.2 → 52.3 Steps/day: 5932 → 7888 % Active: 31% → 32%	Intervention showed significantly higher gains in MET-min, steps, and % active compared with control (Significant ↑)	*p* < 0.05	Multicomponent digital support significantly enhanced PA and self-efficacy
Ansari K, 2023 [52]	55 / 55	3	MET-min/week; Intensity-specific % Active	IPAQ	Text: 350 → 555 Social: 81 → 1631	Minimal change	Social networking group showed much larger PA gains than SMS/text messaging (Significant ↑)	*p* < 0.001	Mobile social networking produced much larger gains than text messaging

**Table 4:** Summary of peer-led / chw-facilitated interventions and their impact on physical activity in low- and middle-income countries (LMICs) included in the review (*n* = 8)

Study ID	Sample Size (intervention /control)	Follow-up (months)	PA measure(s)	Method of reporting PA	Intervention effect (baseline → endline)	Control effect (baseline → endline)	Reported comparative effect (intervention vs. control)	Significance	Conclusions
Pazoki R, 2007 [42]	179 / 179	2	Intensity-specific MET-min/week	Self-reported BRFSS; USA/CDC (2002), and the Countrywide Integrated NCD Intervention program questionnaire	Moderate: 18 → 116 min/wk Vigorous: 4 → 21 min/wk	Moderate: 27 → 37 min/wk Vigorous: 4 → 3 min/wk	Intervention group showed large ↑ in both moderate and vigorous PA compared with control (Significant ↑)	*p* < 0.03; 0.001	Volunteer-led lifestyle program significantly ↑ PA and reduced BP
Chao, 2012 [43]	1163 / 1198	18	PA duration (min/week)	Self reported questionnaire.	119 → 270 min/wk	104 → 169 min/wk	Intervention ↑ PA by ~ 100 min/wk vs. control (Significant ↑)	*p* < 0.001	Community-based health management significantly ↑ PA among elderly.
Anthony, 2015 [53]	6694 / 5442	18 & 24	% Active ≥ 30 min/day (self-report); PA MET-min/day	IPAQ	% Active: 30.8% → 38.6% (+ 7.8%) METS min/day: 217 → 164 (–53)	% Active: 36.2% → 40.9% (+ 4.7%) MET-min/day: 259 → 168 (–91)	Both groups improved slightly in % Active, but overall no significant between-group difference in PA outcomes (No effect)	*p* = 0.078	Multi-country workplace/CHW programs showed modest trends but no significant PA effect
Mouodi S, 2019 [49]	high-intensity: 106, low-intensity: 98 control: 97	4	Physical activity subscale of Healthy Lifestyle Behaviors Questionnaire	Self reported questionnaire.	High-intensity: 13.0 → 17.1 (+ 4.1) Low-intensity: 13.4 → 15.8 (+ 2.4)	Control: 12.6 → 14.9 (+ 2.3)	Intervention groups showed significantly greater ↑ in PA score vs. control (Significant ↑)	Between groups: *p* = 0.015 Repeated measures: *p* = 0.001	Group-based PA + nutrition sessions significantly improved PA scores, especially in high-intensity arm
Meurer, 2019 [48]	135 / 156	3	Sedentary time (min/day); Domain specific PA (min/day)	accelerometers	Sedentary: 391 → 389 (NS) Light PA: 522 → 519 (NS) MVPA: 47 → 53 (+ 6, significant) Total PA: 569 → 571 (NS)	Sedentary: 370 → 380 (NS) Light PA: 544 → 533 (NS) MVPA: 46 → 44 (NS) Total PA: 590 → 580 (NS)	Only MVPA improved significantly in intervention vs. control (*p* = 0.01); other domains NS (Mixed/Partial)	MVPA: *p* = 0.01 Others: NS	VAMOS program improved MVPA, no effect on sedentary, light, or total PA
Mathews E, 2021 [61]	200 / 201	13	% Active (≥ 600 MET-min/wk); MET-min/week	GPAQ	% Active: 0% → 58.5% (4 mo) → 48.5% (7 mo) → 29.6% (13 mo) MET-min/wk: 84 → 1159 (4 mo), 707 (7 mo), 526 (13 mo)	% Active: 0% → 10% (4 mo) → 6% (7 mo) → 0.6% (13 mo) MET-min/wk: 121 → 202 (4 mo), 168 (7 mo), 95 (13 mo)	Large ↑ in PA at 4 mo sustained partially up to 13 mo, but with clear waning of effect (Mixed/Partial over long term)	*p* < 0.001	Peer-support intervention ↑ PA substantially at 4 mo but effect declined over time, though remained above control
Eze II, 2021 [62]	188/188	6	Self-reported practices	IPAQ	Adequate PA: 49/188 (26.1%) → 69/183 (37.7%); p-value: <0.0001	Adequate PA: 57/188 (30.3%) → 48/180 (26.7%) (− 3.6 pp)	Between group Adequate PA χ² = 7.92.	*P* = 0.02	intervention led increased adequate PA compared with control
Li et al.,2023 [44]	240/271	24	Leisure-time PA; household PA; work-related PA; total PA; % meeting WHO PA recommendations	Self-reported using PA scale for the elderly;	Leisure-time PA (PASE): ↑ at 4–8 weeks; attenuated at 6 and 12 months; partially sustained at 24 months. % meeting WHO PA recommendations: ↑ at 4–8 weeks; NS at 6–12 months; ↑ again at 24 months. Leisure-time sitting: ↓ at 4 weeks (− 41.7 min/day) and 8 weeks (− 36.3 min/day); NS at 6–12 months; ↓ at 24 months (− 29.4 min/day).	No meaningful change in PA or sedentary time across follow-up.	Between-group reductions in leisure-time sitting at 4 weeks, 8 weeks, and 24 months (− 29 to − 42 min/day).	*P* < 0.05 (sedentary behavior outcomes)	Intervention significantly reduced leisure-time sedentary behaviour compared with control; PA gains attenuated over time indicating waning effect.

**Table 5:** Summary of environmental /infrastructure-based interventions and their impact on physical activity in low- and middle-income countries (LMICs) included in the review (*n* = 6)

Study ID	Sample size (intervention /control)	Follow-up (months)	PA measure(s)	Measurement of PA	Intervention effect (baseline → endline)	Control Effect (baseline → endline)	Reported comparative effect (intervention vs. control)	Significance	Conclusions
Rabiei K, 2010 [63]	2,400–6,200 adults per survey wave from 2 communities; sample size varied by year.	Repeated cross-sectional	intensity and duration of each type of PA in leisure time, daily transportation, and total daily PA	Baecke physical activity questionnaire	Leisure-time PA declined (Females: 3169 → 1206 MET-min/day, *p* < 0.0001; Males: 3006 → 1193 MET-min/day, *p* = 0.05). Transportation PA also declined. (Females:3167 →1202; *p* = 0.60 Males: 2954→ 1176; *p* = 0.06)	Leisure-time PA declined (Females: 3220 → 1209 MET-min/day, *p* < 0.0001; Males: 3117 → 1189 MET-min/day, *p* < 0.0001) Transportation PA also declined (Females: 3211 → 1209 MET-min/day, *p* < 0.0001; Males: 3079 → 1186 MET-min/day, *p* < 0.0001)	Intervention communities showed smaller declines in leisure-time and transport PA than controls	Mixed (domain- and sex-specific effects)	Community-wide interventions slowed PA decline but did not increase absolute PA levels.
Lv J, 2014 [55]	1016/1000	Cross-sectional	PA MET-min/Week PA engagement	IPAQ-SF	MET-min/week: 1204 (495–2373) → 1386 (693–2457)	MET-min/week: 918 (398–1836) → 924 (438–1980)	higher engagement with intervention activities and higher PA level in intervention areas vs. comparison areas at follow-up.	Within-intervention change: *p* = 0.023; Control: *p* = 0.201; Between-area (baseline & follow-up): *p* < 0.001	CBI achieved high engagement and higher endline physical activity participation compared with controls.
Simões EJ, 2017 [65]	9350 surveyed	36	% active in LTPA	IPAQ	Women: ≥3 yrs city exposure OR 1.46 (*p* = 0.006); Participation: OR 10.35 for current > 6 mo; Knowledge OR: 1.50	Reference = no AC-P exposure/never participated	Significant ↑ in LTPA with longer exposure, participation, and awareness (Significant ↑)	*p* < 0.001	Large-scale city program (AC-P) ↑ population-level LTPA, esp. women; equity benefits noted
Baldovino CL, 2023 [56]	825/854	24	Walking prevalence (%); MVPA (min/day)	questionnaires and accelerometers.	Walking: 40.5% → 51.6% Accelerometer MVPA stable ↑ MVPA in males (OR 2.7, *p* = 0.033); ↓ in females in small parks (OR 0.4, *p* = 0.019)	Similar small ↑ walking; no sig. change leisure or MVPA	No significant effect on MVPA (Mixed/Partial)	Transport *p* = 0.38; Leisure *p* = 0.26; MVPA *p* = 0.41; Park effects mixed	Urban renewal & cable-car program ↑ walking, but no effect on MVPA
Torres A, 2013 [64]	1000 Ciclovía / 1000 Cicloruta	Cross-sectional	% Meeting PA guidelines (LTPA vs. transport); Odds ratios	IPAQ	Ciclovía: 59.5% met LTPA recommendation; regular users OR = 1.7; vigorous PA OR = 4.9 Cicloruta: 70.5% met transport PA recommendation; regular users OR = 10.2	N/A	Both programs showed high compliance with PA guidelines in their respective domains (LTPA vs. transport) (Significant ↑)	All significant (*p* < 0.001)	Population-wide programs promoted PA at scale, with complementary effects (LTPA vs. transport), plus added social capital and equity benefits
Cheah YX., 2024 [57]	84/84	6	Total and domain-specific PA (IPAQ-long; MET-min/week), step counts (pedometer; steps/week), physical activity self-regulation (PASR-12 scale)	IPAQ long form; pedometer; PASR-12 Scale	Increases observed in step counts total physical activity (MET-min/week), housework-related PA, and PA self-regulation.	No significant change	Significant improvement in step counts and housework-related PA; no significant difference in total MET-min/week.	Not significant	The intervention improved PA self-regulation, step counts, and selected PA outcomes within the intervention group; however, between-group effects were mixed with small effect sizes.

MVPA: Moderate or vigorous physical activity. ↑= increase

### Secondary Outcomes

14 studies assessed secondary outcomes (Supplementary material 3b). Across the 12 studies reporting BMI, within-group reductions ranged from − 0.2 to − 1.5 kg/m²; however, statistically significant between-group differences were less consistent [43, 49]. Comparatively smaller reductions in BMI (< 1 kg/m²) were reported in 4 studies [46, 51, 52, 60]. Waist circumference reductions ranged from 1.2 to 4.1 cm, although effect sizes and statistical significance varied. Waist-to-Hip Ratio changes were small and not clinically substantial across all studies. BF% changes were reported in only 3 studies, showing small declines (1–2%, nonsignificant in some). BP improvements were noted in Mouodi et al. and Chao et al., with systolic/diastolic reductions of 3–9 mmHg [43, 49]. Peyman et al. also reported modest BP improvements [58]. Fasting Blood Glucose was only assessed in 2 studies, with a reduction of 5–10 mg/dL over 3–6 months [42, 49]. 

## Discussion

In this systematic review, 24 CBI studies were identified, grouped into three broad formats: digital/technology-assisted approaches, Peer-led/CHW-based interventions, and Environmental restructuring. Overall, the CBIs in LMICs demonstrated measurable improvements in PA and related outcomes. A major constraint observed during the review was the inconsistency in measuring and reporting PA across studies. Moreover, substantial variations in intervention formats, outcome definitions, follow-up durations, and analytical methods precluded pooling of results and limited confidence in comparing the effectiveness across formats. Across the included studies, subgroup patterns derived from the descriptive heat maps should be interpreted cautiously, given the small number of studies contributing to several categories. Within these constraints, digital interventions and short-to-medium-duration programs were more frequently associated with statistically significant improvements in PA. In contrast, PA/SB-focused and broader lifestyle interventions showed comparable effects. These observations are descriptive rather than comparative and do not indicate the superiority of specific intervention types; instead, they highlight where the evidence appears more consistent and where further targeted research is needed. We identified only two studies that also focused on reducing SB in addition to improving PA, underscoring an important area for future research. Unlike prior reviews that primarily synthesized effect sizes or short-term behavioral outcomes, the present study reviewed CBI in LMICs through a systems-adoption lens [14–16]. The focus on delivery formats, duration, fidelity structures, human resource models, and methodological robustness can help identify intervention types that are structurally compatible with public-sector implementation rather than merely statistically effective under controlled conditions.

A key finding from this review is that most CBIs reported improvements in at least one PA outcome, despite substantial variation in design, delivery, and intervention intensity. While this consistency in direction suggests potential benefit across diverse LMIC contexts, confidence in interpretation is constrained by generally weak study quality and marked heterogeneity in outcome measurement and reporting. Similar limitations have been observed previously, where heterogeneity in intervention content, settings, and outcomes necessitated a descriptive synthesis rather than a meta-analysis, despite a single-country context [66]. However, heterogeneity might not be limited to intervention in LMICs but might be a structural feature of complex, community-based behavioral interventions delivered across real-world settings [17]. 

Across intervention formats, digital interventions more frequently reported statistically significant improvements in PA; however, this pattern should be interpreted cautiously, given the small number of studies per subgroup, reliance on self-reported PA outcomes, and generally shorter follow-up periods. The more positive results on PA in this format may partly reflect their alignment with younger, mobile, and technologically engaged populations, who are more likely to respond to app-based prompts, self-monitoring tools, and real-time feedback mechanisms. In fact, several of the included studies recruited students or working-age adults with smartphone access, suggesting that digital uptake may be facilitated in tech-savvy cohorts [67]. Digital interventions are also relatively less resource-intensive once developed, requiring fewer ongoing human resources compared with facilitator-led or CHW-based models. However, this apparent efficiency may mask equity considerations, as sustained engagement, digital literacy, and access to smartphones or internet connectivity are not uniformly distributed across LMIC populations [68]. While some of the included studies using this format were short-term, meta-analytic evidence also indicates that app-based interventions often show modest but statistically significant short-term gains, consistent with engagement-driven behavior change that may attenuate over longer follow-up [69]. 

Peer-led and CHW-facilitated interventions commonly reported indicators of participant engagement and short-term improvements in PA, but evidence of sustained effects beyond the active intervention period was limited, with several studies reporting attenuation over time [70]. Given the mixed findings in this review and available global evidence, these approaches should be interpreted as potentially useful but not yet consistently effective for sustained PA improvement in LMICs [45, 61, 71]. Likewise, the environmental or infrastructural interventions showed more mixed effects, with impacts that appeared more context-dependent and less immediately detectable than those of digital interventions. Having said that, the intervention formats identified in this review reflect those evaluated in LMIC studies and do not capture the full spectrum of physical activity–promoting strategies. Evidence from reviews conducted predominantly in HICs highlights additional approaches, such as primary care referral, mass media campaigns, and active transport or urban design interventions, that were largely absent from the LMIC literature [72–74]. 

We observed that intervention duration was associated with differences in reported PA outcomes. Short- and medium-term programs (≤ 12 months) more frequently demonstrated statistically significant improvements in PA, whereas longer-duration interventions (> 12 months) showed more mixed or partial effects across outcomes. The lower effectiveness of long-term interventions looks counterintuitive but likely reflects differences in intervention type and evaluation context rather than reduced effectiveness of longer engagement per se. In this review, longer-duration interventions were disproportionately environmental or infrastructural and were commonly evaluated as natural experiments. While these studies lack randomization and are susceptible to selection bias, they offer advantages in terms of external validity, scalability, and policy relevance [75]. These interventions target upstream determinants of PA, such as urban design, transportation infrastructure, and environmental support, and are therefore well-suited to capturing context-level change. However, behavior change through such pathways is indirect, depends on uptake and sustained exposure, and is typically assessed over longer periods, increasing the likelihood of mixed or domain-specific effects. While the observed pattern is consistent with other reviews reporting stronger short-term gains, contrasting evidence emerges from meta-analyses that are restricted to trials with harmonized objective outcomes and longer follow-up. For example, Gasana et al. found small effects on steps and MVPA at follow-up periods of 24 months or longer, with lower heterogeneity than is typically seen in reviews combining diverse self-reported outcomes [61, 76, 77]. Therefore, it is recommended that the intervention duration should not be interpreted in isolation from intervention format and evaluation approach.

We observed that both PA/SB-focused and broader lifestyle interventions were associated with improvements in PA outcomes, with no clear differences in effectiveness when examined descriptively by intervention complexity. Broader lifestyle interventions that combined PA with nutrition education or behavioral counseling reported improvements in PA and secondary outcomes, such as BMI and blood pressure [43, 49]. However, evidence from outside this review suggests that addressing multiple behavioral targets simultaneously may dilute effects on individual behaviors such as PA, particularly in resource-constrained settings [78]. In our synthesis, simpler PA/SB-focused interventions—especially digital or pedometer-based approaches—were more frequently associated with statistically significant short-term improvements in PA; however, these findings were largely based on self-reported outcomes and shorter follow-up durations [79]. The review highlights the importance of distinguishing between short-term effectiveness and long-term sustainability when assessing intervention complexity in LMIC contexts, as broader lifestyle intervention-based approaches may be better suited to addressing the structural determinants of other behavioral risk factors, including PA, despite less consistent short-term effects [17]. Interpretation of intervention complexity is further limited by how behavioral theory was reported across studies. Although several studies referenced behavioral theories, reporting was inconsistent and rarely linked to specific intervention components or measurable outcomes. As theory use was neither operationalized nor examined analytically, its contribution to the observed effects cannot be inferred, which also represents a key limitation of the current evidence base.

Across studies, substantial variability was also observed in who delivered the intervention, the number of providers involved, and the extent of training and fidelity monitoring. While the digitalization of adherence monitoring allows standardization of content delivery, most such studies were rated weak on the EPHPP, suggesting that automation alone does not guarantee methodological robustness. Overall, fidelity assessment was most explicitly documented in digital formats (through usage tracking), but depth of implementation monitoring appeared stronger in structured community-based models, highlighting a trade-off between scalability and implementation transparency. In the long run, it can be inferred that clearly defined provider roles, structured training, and embedded monitoring mechanisms are more critical for intervention quality and scalability than delivery format alone.

Although SB was included in the review objectives, only 2 studies explored SB alongside PA. Most interventions focused exclusively on increasing PA, while assuming that changes in PA also translate into meaningful reductions in sedentary time. This gap is notable, given strong evidence, largely from HICs, that SB is independently associated with cardiometabolic risk and all-cause mortality, and that its adverse effects are not fully mitigated by higher PA levels [78, 80–83]. The absence of SB-focused interventions in LMICs, therefore, represents a critical evidence gap and constrains interpretation of CBI effects on overall movement behaviors. Future studies should explicitly conceptualize SB as a distinct behavioral target, consistent with WHO GAPPA’s dual emphasis on increasing PA and reducing SB, and evaluate context-appropriate strategies such as reducing prolonged sitting in workplaces or embedding movement opportunities into daily routines [6, 78]. 

This study has key strengths and limitations that should be acknowledged. A major strength of this review lies in its comprehensive and inclusive search strategy, spanning peer-reviewed and grey literature across diverse LMIC settings and including a broad range of study designs, including RCTs, quasi-experimental studies, and natural experiments, thereby reflecting real-world implementation contexts. Study quality was systematically assessed using established tools, enabling a transparent evaluation of methodological limitations. However, this review is also subject to limitations. Substantial heterogeneity in intervention types, outcome measures, and follow-up durations precluded meta-analytic synthesis and limited cross-study comparability. This limitation reflects a persistent constraint in PA intervention literature rather than an issue unique to this review. Previous reviews have similarly reported that reliance on self-reported PA measures, variation in outcome definitions and reporting units, and limited use of objective assessment tools reduce comparability across studies and constrain synthesis [16, 41, 84]. Most included studies were rated as methodologically weak, commonly due to short follow-up periods, high attrition, limited blinding, and reliance on self-reported PA measures rather than objective assessment tools such as accelerometers. Reporting on implementation processes, particularly regarding fidelity, cost-effectiveness, and policy integration, was inconsistent or absent in most studies, thereby constraining inferences about the long-term impact and scalability. The restriction to English-language publications may have introduced language bias, particularly because some community-based interventions from LMICs may be published in local or regional languages and therefore may not have been captured in this review.

Despite these limitations, several policy-relevant implications emerge from the findings. CBIs show potential to contribute to PA promotion in LMICs, and align with priorities outlined in WHO’s GAPPA, particularly its emphasis on creating an active people, an active society, an active environment, and an active system through scalable, community-based, multisectoral approaches [40]. Interventions that were low-cost, locally adaptable, and delivered through existing community or digital infrastructures were more frequently evaluated and reported short-term improvements in PA. However, the limited and inconsistent reporting of fidelity, sustainability, and economic considerations highlights a critical gap in implementation. Although sustainability was not formally assessed, frameworks such as the Program Sustainability Assessment Tool highlight the importance of environmental support, partnerships, and funding stability, underscoring the need to embed sustainability mechanisms during program design rather than relying on time-limited delivery [85, 86]. Future LMIC research should prioritize rigorous study designs with longer follow-up periods, embedded process evaluations, and clearer reporting of implementation fidelity. Equally important is to standardize PA and SB measurement by specifying the domains assessed, such as leisure-time, transport-related, occupational, household PA, and sedentary time, and by using comparable reporting units such as MET-min/week, MVPA minutes/week, steps/day, and sitting time. Where feasible, device-based measures should be incorporated to complement self-reported data and reduce measurement bias. Such harmonized measurement and reporting would improve comparability across LMIC studies, support pooled analyses, and generate stronger evidence for scale-up. Where feasible, evaluations should also attempt to examine reach, acceptability, and maintenance to better inform integration into routine health and community systems. Targeted exploration of underrepresented populations and hybrid delivery models may be warranted, but these approaches require empirical testing rather than assumptions.

## Conclusion

Our systematic review indicates that CBIs can improve physical activity among adults in LMICs. Only two studies targeted SB alongside PA, underscoring a major evidence gap. Although exploratory subgroup patterns suggest that certain intervention types, particularly digital and shorter-duration programs, may be more consistently associated with positive physical activity outcomes, the limited number of studies and heterogeneity across interventions preclude firm conclusions regarding comparative effectiveness. PA/SB-focused and broader lifestyle interventions demonstrated similar proportions of significant, mixed, and null effects. The impact of duration on effectiveness looks counterintuitive but underscores the need for more rigorous evaluation of longer-duration interventions. Strengthening the evidence base will require more rigorous and sustained evaluations, particularly for sedentary behavior and long-term intervention effects.

## Key References

- Nash EA, Critchley JA, Pearson F, Awad SF, Abu-Raddad LJ, Abu-Hijleh FM, et al. A systematic review of interventions to promote physical activity in six Gulf countries. PLoS One. 2021;16(10 October):1–20
  - ○ A prior systematic review focused on six Gulf countries reported heterogeneous intervention effects and limited generalisability beyond that region (Nash et al., 2021), underscoring the need for a comprehensive synthesis across diverse LMIC settings, particularly CBI targeting PA and/or SB.
- Li N, Ye Q, Deng Q, Wang Y, Hu J, Li X, et al. Physical Activity Intervention for Leisure-Time Activity Levels among Older Adults: A Cluster Randomized Trial. JAMA Netw Open. 2023;6(9):E2333195
  - ○ One of the very few cluster-randomized trials from LMICs, done in older adults, that targeted SB, and demonstrated significant short-term gains in leisure-time PA and reductions in SB, though effects attenuated over extended follow-up.
- Baldovino-Chiquillo L, Sarmiento OL, O’Donovan G, Wilches-Mogollon MA, Aguilar AF, Florez-Pregonero A, et al. Effects of an urban cable car intervention on physical activity: the TrUST natural experiment in Bogotá, Colombia. Lancet Glob Heal. 2023;11(8):e1290–300
  - ○ Recent urban natural experiments in LMICs demonstrate that infrastructure interventions, such as transport-linked cable car systems, can modify walking behavior, although effects on moderate-to-vigorous physical activity remain modest.

## Supplementary Information

Below is the link to the electronic supplementary material.

Supplementary Material 1: Search strategy for retrieving studies from search engines (PubMed, Embase, Scopus)

Supplementary Material 2: Data extraction sheet

Supplementary Material 3: Details of intervention delivery personnel, number of providers, and provider training. Summary of the secondary outcomes measured in the included studies

## Data Availability

No datasets were generated or analysed during the current study.
