# Supplementary material for: A Systematic Review of Community-based Interventions to Promote Physical Activity or Reduce Sedentary Behavior among Adults in Low- and Middle-Income Countries
Source: Curr Obes Rep. 2026 Jul 22;15(1):61. doi: 10.1007/s13679-026-00741-4 (PMC13391703; doi:10.1007/s13679-026-00741-4)
Supplement: Supplementary file 1 — Supplementary Material 1: Search strategy for retrieving studies from search engines (PubMed, Embase, Scopus) [file 13679_2026_741_MOESM1_ESM.docx]

**Annexure 1**

**A systematic review of the community-based interventions to promote physical activity or reduce sedentary behaviour among adults in low- and middle-income countries.**

**Database:**

- **Medline (via Pubmed);** Embase (via Ovid), Science Direct, and Scopus (including Cochrane Library, Medline)

1. **PUBMED search strategy:** 2835 results

1. (exert*[tw]) OR (exercis*[tw]) OR (physical activi*[tw]) OR (physical fitn*[tw]) OR (sport*[tw]) OR (motor activ*[tw])

2. (walk*[tw]) OR (jog[tw]) OR (swim*[tw]) OR (cycl*[tw]) OR (run[tw]) OR (jogging[tw]) OR (running[tw])

3. (weight lift*[tw]) OR (strength train*[tw]) OR (resistance train*[tw]) OR (circuit train*[tw]) OR (weight train*[tw]) OR (aerobic train*[tw]) OR (cardio train*[tw]) OR (aerob*[tw]) OR (HIIT[tw]) OR (biking[tw])

4. "exercise"[MeSH Terms] OR "physical education and training"[MeSH Terms] OR "education"[MeSH Terms] OR "physical fitness"[MeSH Terms] OR "sports"[MeSH Terms] OR "physical exertion"[MeSH Terms] OR “motor activity”[MeSH Terms]

5. (((((((((Behavior, Sedentary) OR (Sedentary Behaviors)) OR (Sedentary Lifestyle)) OR (Lifestyle Sedentary)) OR (Physical Inactivity)) OR (Inactivity Physical)) OR (Lack of Physical Activity)) OR (Sedentary Time)) OR (Sedentary Times)) OR (Time, Sedentary)

**6. 1 OR 2 OR 3 OR 4 or 5**

7. (randomized controlled trial[pt] OR controlled clinical trial[pt] OR randomized[tiab] OR randomized [tiab] OR placebo[tiab] OR clinical trials as a topic[mesh:noexp] OR randomly[tiab] OR trial[ti] NOT (animals[mh]))

8. (epidemiologic studies[MeSH Terms]) OR (case control studies[MeSH Terms]) OR (cohort studies[MeSH Terms]) OR (cross-sectional stud*[MeSH Terms]) OR (case control[tw]) OR (cohort stud*[tw]) OR (cohort analy*[tw]) OR (follow up stud*[tw]) OR (observational stud*[tw]) OR (longitudinal[tw]) OR (retrospective[tw]) OR (cross sectional[tw]) OR (follow-up[tw]) OR (followup[tw])

**9. 7 OR 8**

10. LMICs OR Transitional country OR lower income economy OR underdeveloped economy OR poorer nations OR deprived country

(((((((((((((((((((((((((((((((((((((((((((Developing Country) OR (Developing Nations)) OR (Least Developed Countries)) OR (Country, Least Developed)) OR (Developed Country, Least)) OR (Least Developed Country)) OR (Less-Developed Countries)) OR (Country, Less-Developed)) OR (Less Developed Countries)) OR (Less-Developed Country)) OR (LMICs)) OR (Third-World Countries)) OR (Countries, Third-World)) OR (Country, Third-World)) OR (Third World Countries)) OR (Third-World Country)) OR (Third-World Nations)) OR (Nation, Third-World)) OR (Third World Nations)) OR (Third-World Nation)) OR (Under-Developed Countries)) OR (Country, Under-Developed)) OR (Under Developed Countries)) OR (Under-Developed Country)) OR (Under-Developed Nations)) OR (Nation, Under-Developed)) OR (Under Developed Nations)) OR (Under-Developed Nation)) OR (Low and Middle Income Countries)) OR (Less-Developed Nations)) OR (Less Developed Nations)) OR (Less-Developed Nation)) OR (Nation, Less-Developed)) OR (Low Income Countries)) OR (Country, Low Income)) OR (Low Income Country)) OR (Middle Income Countries)) OR (Countries, Middle Income)) OR (Country, Middle Income)) OR (Middle Income Country)) OR (Lower-Middle-Income Country)) OR (Country, Lower-Middle-Income)) OR (Lower Middle Income Country)) OR (Lower-Middle-Income Countries)

11. 6 AND 9 AND 10 (**Final SEARCH Strategy):**

(((((((exert*[tw]) OR (exercis*[tw]) OR (physical activi*[tw]) OR (physical fitn*[tw]) OR (sport*[tw]) OR (motor activ*[tw])) OR ((walk*[tw]) OR (jog[tw]) OR (swim*[tw]) OR (cycl*[tw]) OR (run[tw]) OR (jogging[tw]) OR (running[tw]))) OR ((weight lift*[tw]) OR (strength train*[tw]) OR (resistance train*[tw]) OR (circuit train*[tw]) OR (weight train*[tw]) OR (aerobic train*[tw]) OR (cardio train*[tw]) OR (aerob*[tw]) OR (HIIT[tw]) OR (biking[tw]))) OR ("exercise"[MeSH Terms] OR "physical education and training"[MeSH Terms] OR "education"[MeSH Terms] OR "physical fitness"[MeSH Terms] OR "sports"[MeSH Terms] OR "physical exertion"[MeSH Terms] OR "motor activity"[MeSH Terms])) OR ((((((((((Behavior, Sedentary) OR (Sedentary Behaviors)) OR (Sedentary Lifestyle)) OR (Lifestyle Sedentary)) OR (Physical Inactivity)) OR (Inactivity Physical)) OR (Lack of Physical Activity)) OR (Sedentary Time)) OR (Sedentary Times)) OR (Time, Sedentary))) AND (((randomized controlled trial[pt] OR controlled clinical trial[pt] OR randomized[tiab] OR randomized [tiab] OR placebo[tiab] OR randomly[tiab] OR trial[ti] NOT (animals[mh]))) OR ((epidemiologic studies[MeSH Terms]) OR (case control studies[MeSH Terms]) OR (cohort studies[MeSH Terms]) OR (cross-sectional stud*[MeSH Terms]) OR (case control[tw]) OR (cohort stud*[tw]) OR (cohort analy*[tw]) OR (follow up stud*[tw]) OR (observational stud*[tw]) OR (longitudinal[tw]) OR (retrospective[tw]) OR (cross sectional[tw]) OR (follow-up[tw]) OR (followup[tw])))) AND ((((((((((((((((((((((((((((((((((((((((((((Developing Country) OR (Developing Nations)) OR (Least Developed Countries)) OR (Country, Least Developed)) OR (Developed Country, Least)) OR (Least Developed Country)) OR (Less-Developed Countries)) OR (Country, Less-Developed)) OR (Less Developed Countries)) OR (Less-Developed Country)) OR (LMICs)) OR (Third-World Countries)) OR (Countries, Third-World)) OR (Country, Third-World)) OR (Third World Countries)) OR (Third-World Country)) OR (Third-World Nations)) OR (Nation, Third-World)) OR (Third World Nations)) OR (Third-World Nation)) OR (Under-Developed Countries)) OR (Country, Under-Developed)) OR (Under Developed Countries)) OR (Under-Developed Country)) OR (Under-Developed Nations)) OR (Nation, Under-Developed)) OR (Under Developed Nations)) OR (Under-Developed Nation)) OR (Low and Middle Income Countries)) OR (Less-Developed Nations)) OR (Less Developed Nations)) OR (Less-Developed Nation)) OR (Nation, Less-Developed)) OR (Low Income Countries)) OR (Country, Low Income)) OR (Low Income Country)) OR (Middle Income Countries)) OR (Countries, Middle Income)) OR (Country, Middle Income)) OR (Middle Income Country)) OR (Lower-Middle-Income Country)) OR (Country, Lower-Middle-Income)) OR (Lower Middle Income Country)) OR (Lower-Middle-Income Countries))

1. **EMBASE Sources: Embase, MEDLINE, Preprints;**

Embase session results-

**#1** ('physical activity, capacity and performance'/exp OR 'physical activity, capacity and performance') AND ([cochrane review]/lim OR [systematic review]/lim OR [meta analysis]/lim OR [randomized controlled trial]/lim OR 'controlled clinical trial'/de) AND ([article]/lim OR [article in press]/lim OR [conference abstract]/lim OR [conference paper]/lim OR [data papers]/lim OR [erratum]/lim OR [review]/lim OR [preprint]/lim) AND ([adolescent]/lim OR [adult]/lim OR [young adult]/lim OR [middle aged]/lim OR [aged]/lim OR [very elderly]/lim) AND [humans]/lim AND [english]/lim AND ([embase]/lim OR [medline]/lim) AND [2000-2024]/py AND [medline]/lim

**#2 1213**

('sedentary lifestyle'/exp OR 'sedentary lifestyle') AND ([cochrane review]/lim OR [systematic review]/lim OR [meta analysis]/lim OR [randomized controlled trial]/lim OR 'controlled clinical trial'/de) AND ([article]/lim OR [article in press]/lim OR [conference abstract]/lim OR [conference paper]/lim OR [conference review]/lim OR [data papers]/lim OR [editorial]/lim OR [erratum]/lim OR [preprint]/lim) AND ([adult]/lim OR [young adult]/lim OR [middle aged]/lim OR [aged]/lim OR [very elderly]/lim) AND [humans]/lim AND ([embase]/lim OR [medline]/lim OR [preprint]/lim) AND [2000-2024]/py AND [medline]/lim

**#3 #1 OR #2 47320**

**#4 113517 Studies**

(((('afghanistan'/exp OR afghanistan OR 'albania'/exp OR albania OR 'algeria'/exp OR algeria OR 'american'/exp OR american) AND ('samoa'/exp OR samoa) OR 'angola'/exp OR angola OR 'armenia'/exp OR armenia OR 'azerbaijan'/exp OR azerbaijan OR 'bangladesh'/exp OR bangladesh OR 'belarus'/exp OR belarus OR 'belize'/exp OR belize OR 'benin'/exp OR benin OR 'bhutan'/exp OR bhutan OR 'bolivia'/exp OR bolivia OR 'bosnia and herzegovina'/exp OR 'bosnia and herzegovina' OR 'botswana'/exp OR botswana OR 'brazil'/exp OR brazil OR 'bulgaria'/exp OR bulgaria OR burkina) AND faso OR 'burundi'/exp OR burundi OR cabo) AND verde OR 'cambodia'/exp OR cambodia OR 'cameroon'/exp OR cameroon OR 'central african republic'/exp OR 'central african republic' OR 'chad'/exp OR chad OR 'china'/exp OR china OR 'colombia'/exp OR colombia OR 'comoros'/exp OR comoros OR 'congo dem. rep.' OR 'congo, republic' OR 'costa rica'/exp OR 'costa rica' OR 'cote d' OR 'cuba'/exp OR cuba OR 'djibouti'/exp OR djibouti OR 'dominica'/exp OR dominica OR 'dominican republic'/exp OR 'dominican republic' OR 'ecuador'/exp OR ecuador OR 'egypt, arab republic' OR 'el salvador'/exp OR 'el salvador' OR 'equatorial guinea'/exp OR 'equatorial guinea' OR 'eritrea'/exp OR eritrea OR 'ethiopia'/exp OR ethiopia OR 'fiji'/exp OR fiji OR 'gabon'/exp OR gabon OR 'gambia, the' OR 'georgia'/exp OR georgia OR 'ghana'/exp OR ghana OR 'grenada'/exp OR grenada OR 'guatemala'/exp OR guatemala OR 'guinea'/exp OR guinea OR 'guinea-bissau'/exp OR 'guinea-bissau' OR 'guyana'/exp OR guyana OR 'haiti'/exp OR haiti OR 'honduras'/exp OR honduras OR 'india'/exp OR india OR 'indonesia'/exp OR indonesia OR 'iran, islamic republic' OR 'iraq'/exp OR iraq OR 'jamaica'/exp OR jamaica OR 'jordan'/exp OR jordan OR 'kazakhstan'/exp OR kazakhstan OR 'kenya'/exp OR kenya OR 'kiribati'/exp OR kiribati OR 'korea, dem. people republic' OR 'kosovo'/exp OR kosovo OR 'kyrgyz republic'/exp OR 'kyrgyz republic' OR 'lao pdr' OR 'iran'/exp OR iran OR 'korea'/exp OR korea OR 'gambia'/exp OR gambia OR 'lebanon'/exp OR lebanon OR 'lesotho'/exp OR lesotho OR 'liberia'/exp OR liberia OR 'libya'/exp OR libya OR 'macedonia, fyr' OR 'madagascar'/exp OR madagascar OR 'malawi'/exp OR malawi OR 'malaysia'/exp OR malaysia OR 'maldives'/exp OR maldives OR 'mali'/exp OR mali OR 'marshall islands'/exp OR 'marshall islands' OR 'mauritania'/exp OR mauritania OR 'mauritius'/exp OR mauritius OR 'mexico'/exp OR mexico OR 'micronesia, federation sts.' OR 'moldova'/exp OR moldova OR 'mongolia'/exp OR mongolia OR 'montenegro'/exp OR montenegro OR 'morocco'/exp OR morocco OR 'mozambique'/exp OR mozambique OR 'myanmar'/exp OR myanmar OR 'namibia'/exp OR namibia OR 'nepal'/exp OR nepal OR 'nicaragua'/exp OR nicaragua OR 'niger'/exp OR niger OR 'nigeria'/exp OR nigeria OR 'pakistan'/exp OR pakistan OR 'palau'/exp OR palau OR 'panama'/exp OR panama OR 'papua new guinea'/exp OR 'papua new guinea' OR 'paraguay'/exp OR paraguay OR 'peru'/exp OR peru OR 'philippines'/exp OR philippines OR 'romania'/exp OR romania OR 'russian federation'/exp OR 'russian federation' OR 'rwanda'/exp OR rwanda OR 'samoa'/exp OR samoa OR 'sao tome and principe'/exp OR 'sao tome and principe' OR 'senegal'/exp OR senegal OR 'serbia'/exp OR serbia OR 'sierra leone'/exp OR 'sierra leone' OR 'solomon islands'/exp OR 'solomon islands' OR 'somalia'/exp OR somalia OR 'south africa'/exp OR 'south africa' OR 'south sudan'/exp OR 'south sudan' OR 'sri lanka'/exp OR 'sri lanka' OR 'st. lucia'/exp OR 'st. lucia' OR 'st. vincent and the grenadines'/exp OR 'st. vincent and the grenadines' OR 'sudan'/exp OR sudan OR 'suriname'/exp OR suriname OR 'swaziland'/exp OR swaziland OR 'syrian arab republic'/exp OR 'syrian arab republic' OR 'tajikistan'/exp OR tajikistan OR 'tanzania'/exp OR tanzania OR 'thailand'/exp OR thailand OR 'timor-leste'/exp OR 'timor-leste' OR 'togo'/exp OR togo OR 'tonga'/exp OR tonga OR 'tunisia'/exp OR tunisia OR 'turkey'/exp OR turkey OR 'turkmenistan'/exp OR turkmenistan OR 'tuvalu'/exp OR tuvalu OR 'uganda'/exp OR uganda OR 'ukraine'/exp OR ukraine OR 'uzbekistan'/exp OR uzbekistan OR 'vanuatu'/exp OR vanuatu OR 'venezuela, rb' OR 'vietnam'/exp OR vietnam OR 'west bank and gaza' OR 'yemen, republic' OR 'zambia'/exp OR zambia OR 'zimbabwe'/exp OR zimbabwe OR 'transitional country' OR 'transitional countries' OR 'lami countries' OR 'lami country' OR lmics OR lmic OR 'third world' OR 'lower income economy' OR 'low income economy'/exp OR 'low income economy' OR 'lower income economies' OR 'low income economies' OR 'middle income economies' OR 'middle income economy'/exp OR 'middle income economy' OR 'underdeveloped economy' OR 'underdeveloped economies' OR 'under developed economies' OR 'under developed economy' OR 'lesser developed economy' OR 'lesser developed economies' OR 'less developed economies' OR 'less developed economy' OR 'developing economies' OR 'developing economy' OR 'poorer world' OR 'poorer populations' OR 'poorer population' OR 'poorer countries' OR 'poorer country' OR 'poorer nations' OR 'poorer nation' OR 'poor world' OR 'poor population' OR 'poor populations' OR 'poor nations' OR 'poor nation' OR 'poor countries' OR 'poor country' OR 'deprived world' OR 'deprived populations' OR 'deprived population' OR 'deprived nation' OR 'deprived nations' OR 'deprived countries' OR 'deprived country' OR 'under served world' OR 'underserved world' OR 'underserved countries' OR 'underserved country' OR 'under served nations' OR 'under served nation' OR 'underserved nation' OR 'underserved nations' OR 'underserved population' OR 'underserved populations' OR 'under served populations' OR 'under served population' OR 'under served countries' OR 'under served country' OR 'low income countries' OR 'low income population'/exp OR 'low income population' OR 'low income populations' OR 'low income nations' OR 'low income country'/exp OR 'low income country' OR 'middle income populations' OR 'middle income population'/exp OR 'middle income population' OR 'middle income nations' OR 'middle income nation' OR 'middle income countries' OR 'middle income country'/exp OR 'middle income country' OR 'underdeveloped world' OR 'underdeveloped populations' OR 'underdeveloped population' OR 'underdeveloped nations' OR 'underdeveloped nation' OR 'under developed world' OR 'under developed nation' OR 'under developed countries' OR 'under developed country'/exp OR 'under developed country' OR 'under developed populations' OR 'under developed population' OR 'under developed nations' OR 'lesser developed world' OR 'lesser developed population' OR 'lesser developed nations' OR 'lesser developed nation' OR 'lesser developed countries' OR 'lesser developed country' OR 'less developed world' OR 'less developed populations' OR 'less developed population' OR 'less developed nations' OR 'less developed nation' OR 'less developed countries' OR 'less developed country'/exp OR 'less developed country' OR 'developing world' OR 'developing populations' OR 'developing population' OR 'developing nations' OR 'developing nation' OR 'developing countries'/exp OR 'developing countries' OR 'developing country'/exp OR 'developing country' OR 'afghanistan'/exp OR 'afghanistan' OR 'eastern europe'/exp OR 'eastern europe' OR 'pacific islands'/exp OR 'pacific islands' OR 'commonwealth of independent states'/exp OR 'commonwealth of independent states' OR 'atlantic islands'/exp OR 'atlantic islands' OR 'central america'/exp OR 'central america' OR 'latin america'/exp OR 'latin america' OR 'south america'/exp OR 'south america' OR 'west indies'/exp OR 'west indies' OR 'caribbean'/exp OR 'caribbean' OR 'asia'/exp OR 'asia' OR 'africa'/exp OR 'africa' OR 'south east asia'/exp OR 'south east asia') AND ([cochrane review]/lim OR [systematic review]/lim OR [meta analysis]/lim OR [randomized controlled trial]/lim OR 'controlled clinical trial'/de) AND ([article]/lim OR [article in press]/lim OR [conference abstract]/lim OR [conference paper]/lim OR [conference review]/lim OR [data papers]/lim OR [editorial]/lim OR [preprint]/lim) AND ([adult]/lim OR [young adult]/lim OR [middle aged]/lim OR [aged]/lim OR [very elderly]/lim) AND [humans]/lim AND ([embase]/lim OR [medline]/lim OR [preprint]/lim) AND [2000-2024]/py AND [medline]/lim

**#5 #3 AND #4 8342 (Final Search strategy)**

1. **Scopus Final search strategy: 4213 DOCUMENTS**

( ( randomized AND controlled AND trial OR controlled AND clinical AND trial OR randomized OR randomized OR placebo OR clinical AND trials OR randomly OR trial AND not AND animals ) OR ( epidemiologic AND studies OR case AND control AND studies OR cohort AND studies OR cross-sectional AND stud* OR case AND control OR cohort AND stud* OR cohort AND analy* OR follow AND up AND stud* OR observational AND stud* OR longitudinal OR retrospective OR cross AND sectional OR follow-up OR followup ) ) AND ( ( TITLE-ABS-KEY ( walk* OR jog OR swim* OR cycl* OR run OR jogging OR running ) ) OR ( TITLE-ABS-KEY ( weight AND lift* OR strength AND train* OR resistance AND train* OR circuit AND train* OR weight AND train* OR aerobic AND train* OR cardio AND train* OR aerob* OR hiit OR biking ) ) OR ( TITLE-ABS-KEY ( exercise OR physical AND education AND training OR education OR physical AND fitness OR sports OR physical AND exertion OR motor AND activity ) ) OR ( TITLE-ABS-KEY ( ( behavior, AND sedentary ) OR ( sedentary, AND behaviors ) OR ( sedentary, AND lifestyle ) OR ( lifestyle, AND sedentary ) OR ( physical, AND inactivity ) OR ( inactivity, AND physical ) OR ( lack AND of AND physical AND activity ) OR ( sedentary AND time ) OR ( sedentary, AND times ) OR ( time, AND sedentary ) ) ) ) AND PUBYEAR > 1999 AND PUBYEAR < 2025 AND ( LIMIT-TO ( AFFILCOUNTRY , "Albania" ) OR LIMIT-TO ( AFFILCOUNTRY , "Algeria" ) OR LIMIT-TO ( AFFILCOUNTRY , "Angola" ) OR LIMIT-TO ( AFFILCOUNTRY , "Argentina" ) OR LIMIT-TO ( AFFILCOUNTRY , "Armenia" ) OR LIMIT-TO ( AFFILCOUNTRY , "Azerbaijan" ) OR LIMIT-TO ( AFFILCOUNTRY , "Bangladesh" ) OR LIMIT-TO ( AFFILCOUNTRY , "Belarus" ) OR LIMIT-TO ( AFFILCOUNTRY , "Benin" ) OR LIMIT-TO ( AFFILCOUNTRY , "Bhutan" ) OR LIMIT-TO ( AFFILCOUNTRY , "Bosnia and Herzegovina" ) OR LIMIT-TO ( AFFILCOUNTRY , "Botswana" ) OR LIMIT-TO ( AFFILCOUNTRY , "Brazil" ) OR LIMIT-TO ( AFFILCOUNTRY , "Burkina Faso" ) OR LIMIT-TO ( AFFILCOUNTRY , "Cambodia" ) OR LIMIT-TO ( AFFILCOUNTRY , "Cameroon" ) OR LIMIT-TO ( AFFILCOUNTRY , "Central African Republic" ) OR LIMIT-TO ( AFFILCOUNTRY , "Chad" ) OR LIMIT-TO ( AFFILCOUNTRY , "China" ) OR LIMIT-TO ( AFFILCOUNTRY , "Colombia" ) OR LIMIT-TO ( AFFILCOUNTRY , "Congo" ) OR LIMIT-TO ( AFFILCOUNTRY , "Costa Rica" ) OR LIMIT-TO ( AFFILCOUNTRY , "Cote d'Ivoire" ) OR LIMIT-TO ( AFFILCOUNTRY , "Cuba" ) OR LIMIT-TO ( AFFILCOUNTRY , "Dominica" ) OR LIMIT-TO ( AFFILCOUNTRY , "Ecuador" ) OR LIMIT-TO ( AFFILCOUNTRY , "Egypt" ) OR LIMIT-TO ( AFFILCOUNTRY , "Ethiopia" ) OR LIMIT-TO ( AFFILCOUNTRY , "Gabon" ) OR LIMIT-TO ( AFFILCOUNTRY , "Gambia" ) OR LIMIT-TO ( AFFILCOUNTRY , "Georgia" ) OR LIMIT-TO ( AFFILCOUNTRY , "Ghana" ) OR LIMIT-TO ( AFFILCOUNTRY , "Grenada" ) OR LIMIT-TO ( AFFILCOUNTRY , "Guatemala" ) OR LIMIT-TO ( AFFILCOUNTRY , "Guinea" ) OR LIMIT-TO ( AFFILCOUNTRY , "India" ) OR LIMIT-TO ( AFFILCOUNTRY , "Indonesia" ) OR LIMIT-TO ( AFFILCOUNTRY , "Iran" ) OR LIMIT-TO ( AFFILCOUNTRY , "Iraq" ) OR LIMIT-TO ( AFFILCOUNTRY , "Jamaica" ) OR LIMIT-TO ( AFFILCOUNTRY , "Jordan" ) OR LIMIT-TO ( AFFILCOUNTRY , "Kazakhstan" ) OR LIMIT-TO ( AFFILCOUNTRY , "Kenya" ) OR LIMIT-TO ( AFFILCOUNTRY , "Kyrgyzstan" ) OR LIMIT-TO ( AFFILCOUNTRY , "Laos" ) OR LIMIT-TO ( AFFILCOUNTRY , "Lebanon" ) OR LIMIT-TO ( AFFILCOUNTRY , "Liberia" ) OR LIMIT-TO ( AFFILCOUNTRY , "Libya" ) OR LIMIT-TO ( AFFILCOUNTRY , "Madagascar" ) OR LIMIT-TO ( AFFILCOUNTRY , "Malawi" ) OR LIMIT-TO ( AFFILCOUNTRY , "Malaysia" ) OR LIMIT-TO ( AFFILCOUNTRY , "Mali" ) OR LIMIT-TO ( AFFILCOUNTRY , "Mauritius" ) OR LIMIT-TO ( AFFILCOUNTRY , "Mexico" ) OR LIMIT-TO ( AFFILCOUNTRY , "Moldova" ) OR LIMIT-TO ( AFFILCOUNTRY , "Mongolia" ) OR LIMIT-TO ( AFFILCOUNTRY , "Montenegro" ) OR LIMIT-TO ( AFFILCOUNTRY , "Mozambique" ) OR LIMIT-TO ( AFFILCOUNTRY , "Myanmar" ) OR LIMIT-TO ( AFFILCOUNTRY , "Namibia" ) OR LIMIT-TO ( AFFILCOUNTRY , "Nepal" ) OR LIMIT-TO ( AFFILCOUNTRY , "Niger" ) OR LIMIT-TO ( AFFILCOUNTRY , "Nigeria" ) OR LIMIT-TO ( AFFILCOUNTRY , "Pakistan" ) OR LIMIT-TO ( AFFILCOUNTRY , "Panama" ) OR LIMIT-TO ( AFFILCOUNTRY , "Papua New Guinea" ) OR LIMIT-TO ( AFFILCOUNTRY , "Peru" ) OR LIMIT-TO ( AFFILCOUNTRY , "Philippines" ) OR LIMIT-TO ( AFFILCOUNTRY , "Rwanda" ) OR LIMIT-TO ( AFFILCOUNTRY , "Senegal" ) OR LIMIT-TO ( AFFILCOUNTRY , "Serbia" ) OR LIMIT-TO ( AFFILCOUNTRY , "Sierra Leone" ) OR LIMIT-TO ( AFFILCOUNTRY , "South Africa" ) OR LIMIT-TO ( AFFILCOUNTRY , "Sudan" ) OR LIMIT-TO ( AFFILCOUNTRY , "Sri Lanka" ) OR LIMIT-TO ( AFFILCOUNTRY , "Syrian Arab Republic" ) OR LIMIT-TO ( AFFILCOUNTRY , "Thailand" ) OR LIMIT-TO ( AFFILCOUNTRY , "Tunisia" ) OR LIMIT-TO ( AFFILCOUNTRY , "Turkey" ) OR LIMIT-TO ( AFFILCOUNTRY , "Ukraine" ) OR LIMIT-TO ( AFFILCOUNTRY , "Uganda" ) OR LIMIT-TO ( AFFILCOUNTRY , "Uzbekistan" ) OR LIMIT-TO ( AFFILCOUNTRY , "Venezuela" ) OR LIMIT-TO ( AFFILCOUNTRY , "Viet Nam" ) OR LIMIT-TO ( AFFILCOUNTRY , "Yemen" ) OR LIMIT-TO ( AFFILCOUNTRY , "Zambia" ) OR LIMIT-TO ( AFFILCOUNTRY , "Zimbabwe" ) ) AND ( LIMIT-TO ( EXACTKEYWORD , "Human" ) OR LIMIT-TO ( EXACTKEYWORD , "Humans" ) ) AND ( LIMIT-TO ( DOCTYPE , "ar" ) OR LIMIT-TO ( DOCTYPE , "cp" ) OR LIMIT-TO ( DOCTYPE , "sh" ) ) AND ( LIMIT-TO ( SRCTYPE , "j" ) ) AND ( LIMIT-TO ( LANGUAGE , "English" ) )
