# Supplementary material for: A Systematic Review of Community-based Interventions to Promote Physical Activity or Reduce Sedentary Behavior among Adults in Low- and Middle-Income Countries
Source: Curr Obes Rep. 2026 Jul 22;15(1):61. doi: 10.1007/s13679-026-00741-4 (PMC13391703; doi:10.1007/s13679-026-00741-4)
Supplement: Supplementary file 3 — Supplementary Material 3: Details of intervention delivery personnel, number of providers, and provider training. Summary of the secondary outcomes measured in the included studies [file 13679_2026_741_MOESM3_ESM.docx]

**Supplementary material 3**

**Supplementary material 3a: Details of intervention delivery personnel, number of providers, and provider training.**

| **Study Id** | **Who delivers the intervention?** | **Number of providers** | **Training of providers** |
| --- | --- | --- | --- |
| Pazoki R, 2007 [36] | Community health volunteers conducted home visits to deliver physical activity interventions through structured educational sessions. | 53 trained community health volunteers provided home-based interventions. | Volunteers underwent extensive workshops on behavior change techniques and motivational interviewing. |
| Chao, 2012 [37] | Community health managers and trained nurses led a community-based health management program for the elderly. | Ten trained community health managers working across multiple urban centers. | Community health managers received structured training in elderly health management, lifestyle coaching, and chronic disease prevention. |
| Gholamnia SZ, 2017 [39] | Multimedia software specialists and health educators facilitated a TPB-based digital intervention promoting women's physical activity. | A team of four multimedia developers and health promotion specialists. | Multimedia specialists underwent specialized training in digital behavior change interventions and TPB applications. |
| Memon, 2018 [40] | Physiotherapists and research assistants supervised an incentive-based physical activity program for female medical students. | Two physiotherapists and one research assistant per intervention group. | Physiotherapists and research assistants attended workshops on motivational incentives and structured exercise programming. |
| Mui et al, 2018 [41] | Automated telephone counseling system (TLC-PA-China); no human counselor involved | Not applicable (fully automated system) | Not applicable (automated delivery); system scripts developed and culturally adapted by research team |
| Meurer, 2019 [42] | Trained physical education professionals facilitated the VAMOS strategy in community health settings. | One primary physical educator per intervention site, supported by public health staff. | Physical educators received standardized training on the VAMOS strategy and principles of health promotion. |
| Mouodi S, 2019 [43] | Medical doctors, nutritionists, and trained health educators facilitated group sessions and individual consultations. | Three-member team including a medical doctor, MSc in nutrition, and Ph.D. in clinical nutrition. | All educators underwent structured training in nutrition, exercise science, and behavioral counseling before delivering the intervention. |
| Hidrus A, 2020 [44] | Health professionals, including exercise physiologists and medical researchers, delivered Brain Breaks video-based interventions. | A team of five professionals, including exercise physiologists and behavioral researchers. | Providers received training in digital physical activity interventions and behavioral motivation strategies. |
| Alsaleh E, 2023 [45] | A trained public health educator with expertise in behavioral interventions and exercise counseling. | One primary provider with support from assistant educators. | Providers received a 2-week training on motivational interviewing, goal-setting strategies, and structured physical activity programs. |
| Ansari K, 2023[46] | Midwives and reproductive health experts with experience in digital health interventions. | Two midwives and one digital health specialist per intervention group. | Midwives and health professionals were trained in mobile-based health interventions and behavior change techniques. |
| Li et al 2023 [38] | Research team (with peer group leaders facilitating group activities) | NR | Providers reported as trained prior to intervention; details of training not specified |
| Rabiei K, 2010 [57] | multiple executive projects involving public health authorities, municipal agencies, education departments, and other governmental and community stakeholders. | NR | Not reported |
| Torres A, 2013[58] | Urban planners and community health workers promoted physical activity through an infrastructure-based intervention. | Community urban planning teams and health advocates implemented the intervention. | Urban planning professionals were trained in active transportation and public health impact assessments. |
| Lv J, 2014 [49] | local staff of the district Centres for Disease Control and Prevention, doctors of community  health centres, community public health assistants, school doctors and worksite clinic doctors. | NR | Training reported; details not specified |
| Anthony, 2015 [47] | Trained medical professionals and health educators provided physical activity counseling as part of workplace wellness programs. | At least one trained medical professional per participating workplace. | Medical professionals received workplace wellness training, emphasizing behavioral coaching and lifestyle interventions. |
| Baghianimoghaddam, 2016 [48] | University faculty and research staff facilitated a pedometer-based physical activity intervention among university employees. | University-based intervention team comprising researchers and health coaches. | University researchers were trained in behavioral intervention techniques and pedometer-based activity tracking. |
| Simões EJ, 2017[59] | Trained physical educators employed by the Academia das Cidades program delivered physical activity interventions in community settings. | Multiple educators across various community sites, supported by local health departments. | Physical educators received standardized training on structured exercise classes and community engagement strategies. |
| Peyman, 2018 [52] | Public health educators and digital intervention specialists provided digital media-based interventions for increasing physical activity in women. | A digital intervention team comprising six experts in multimedia-based education and public health. | Digital health experts completed training in online health coaching and interactive physical activity content delivery. |
| Pentakota, 2019 [53] | Digital health experts and app developers designed and implemented a mobile app-based physical activity intervention. | A team of app developers, behavioral scientists, and digital health coaches. | App developers were trained in gamification principles, user engagement strategies, and behavioral psychology. |
| Mathew V, 2019 [54] | Trained medical professionals and health educators provided physical activity counseling as part of workplace wellness programs. | At least one trained medical professional per participating workplace. | Medical professionals received workplace wellness training, emphasizing behavioral coaching and lifestyle interventions. |
| Mathews E, 2021[55] | Peer leaders trained in motivational techniques facilitated group-based physical activity interventions. | Multiple peer leaders, one per intervention group, supported by community health organizations. | Peer leaders participated in workshops on physical activity facilitation, group motivation, and community engagement. |
| Eze II, 2021[56] | Public health physician, dietician, and physical fitness counsellor, supported by trained research assistants (Resident doctors) | NR | Training mentioned; details NR |
| Baldovino-CL, 2023 | Health educators and physical activity coaches facilitated guided community walking and exercise sessions. | A team of 10 health educators and physical activity trainers per intervention district. | Health educators were trained in motivational coaching, structured exercise planning, and community engagement. |

**Supplementary material 3b: Summary of the secondary outcomes measured in the included studies.**

| **Study (Year, Country)** | **Secondary Measure(s)** | **Intervention Effect** | **Control Effect** | **Comparative Effect** | **Significance** | **Conclusion** |
| --- | --- | --- | --- | --- | --- | --- |
| **Pazoki R, 2007**[36] | BMI, SBP, DBP, Knowledge score | BMI: 28.0→27.5; SBP: 111.3→110.3; DBP: 68.9→72.6; Knowledge: 17.6→41.1 | BMI: 27.8→28.0; SBP: 111.4→113.6; DBP: 70.0→74.2; Knowledge: 20.5→38.0 | Intervention ↓ SBP and ↑ knowledge significantly; BMI change NS | SBP p=0.04; Knowledge p<0.001 | Volunteer-led program reduced SBP and ↑ knowledge, no effect on BMI |
| **Chao, 2012**[37] | BMI, Waist-to-Hip Ratio | BMI: 24.1→23.5; WHR: 0.89→0.96 | BMI: 23.8→23.3; WHR: 0.88→0.88 | BMI ↓ in both groups; WHR ↑ slightly in intervention | BMI p=0.38; WHR p<0.001 | Community-based health management modestly improved BMI in both groups; WHR changes favored control. |
| **Gholamnia SZ, 2017**[39] | BMI, Waist-to-Hip Ratio, BF% | BMI: 26.2→24.5; WHR: 0.84→0.79; BF%: 28.1→22.8 | BMI stable ~26.4; WHR ~0.84; BF%~28→28 | Significant ↓ in BMI, WHR, and BF% in intervention vs stable control | p<0.001 | WhatsApp-based intervention significantly reduced BMI, waist-hip ratio, and BF% in women. |
| **Memon, 2018**[40] | Weight | Weight: 68.7→68.0 | Weight: 72.1→71.0 | No significant difference | p=0.17 | Incentive-only smartphone app had no significant effect on weight. |
| **Meurer, 2019**[42] | Weight, BMI, Waist | Weight: 76.2→75.4; BMI: 31.3→30.9; WC: 91.3→91.8 | Weight: 74.8→74.8; BMI: 31.1→31.1; WC: 91.4→91.8 | No meaningful difference between groups | NS | VAMOS program improved knowledge/behavior but did not significantly change anthropometric measures. |
| **Mouodi S, 2019**[43] | Weight, BMI, Waist, BF% | High-intensity: Weight 78.2→77.0; BMI 28.3→28.0; WC 94.9→89.3; BF% 31.7→32.7. Low-intensity: Weight 77.8→76.5; BMI 28.2→27.8; WC 94.3→88.6; BF%31.8→31.5. | Control: Weight 78.9→78.2; BMI 28.2→28.2; WC 93.8→90.1; BF%31.2→31.5. | Intervention arms (esp. high-intensity) showed greater ↓ in WC compared with control; BMI changes small; BF% effects inconsistent. | WC p<0.05; BMI/weight NS; BF% NS. | Group PA + nutrition sessions effective in reducing waist circumference; limited impact on BMI/ BF% |
| **Hidrus A, 2020**[44] | BMI, Weight, Waist | BMI: 25.5→25.3; Weight: 66.7→65.8; WC: 91.1→89.6 | BMI: 25.8→26.1; Weight: 67.5→68.0; WC: 90.7→91.3 | Intervention showed small ↓ BMI, weight, WC vs control increase | p=0.05–0.001 | WhatsApp-based “Brain Breaks” improved PA and produced small favorable anthropometric changes. |
| **Alsaleh E, 2023** [45] | Weight, BMI | Weight: 66.2→60.5; BMI: 28.2→25.4 | Weight: 66.2→65.9; BMI: 28.2→28.2 | Significant ↓ in weight and BMI in intervention; no change in control | p<0.05 | Multicomponent digital support ↓ weight and BMI significantly vs control |
| **Ansari K, 2023**[46] | Weight, BMI, Waist, Hip, WHR, BF% | Social group: Weight 76.1→72.7; BMI 29.9→28.6; WC 90.8→87.8; HC 108.4→101.6; WHR 0.85→0.86; BF% 40.3→38.5. Text group: Minimal changes (Weight 76.1→74.8; BMI 31.0→30.7; WC 96.8→94.3; HC 111.7→107.7; BF% 40.3→40.3). | Controls not reported separately (comparison was text vs social). | Social networking group showed significant improvements in weight, BMI, waist, hip, and BF%; text messaging minimal effect. | p<0.001 for most social networking outcomes; NS for text group. | Mobile social networking led to significant anthropometric improvements; SMS-based intervention ineffective. |
| **Anthony, 2015**[47] | BMI (kg/m²), Overweight/Obesity prevalence; Diet (≥5 fruit & veg portions/day); Salt added at table | BMI mean: 23.8→24.4; ≥30 kg/m²: 8.8%→8.1%; ≥25 kg/m²: 25.4%→29.1%; Fruit/veg 2.8→3.3; Salt at table 36.4→41.1 | BMI mean: 23.6→24.2; ≥30 kg/m²: 7.4%→8.1%; ≥25 kg/m²: 23.7%→26.1%; Fruit/veg 2.5→2.8; Salt at table 33.2→44.2 | Diet indicators improved in both groups (↑ fruit/veg; ↓ salt at table), differences generally small; no significant between-group change in BMI. | Fruit/veg p<0.001 (both); Salt p<0.001 (both); BMI mean p=0.089 (ns); BMI ≥30 kg/m² p=0.148 (ns). | Community workplace/CHW program improved diet markers at scale, but no differential effect on BMI/obesity. |
| **Simões EJ, 2017**[59] | Knowledge score (PA, health) | Knowledge score OR = 1.50; more awareness in exposed group | Reference = no AC-P exposure | Significant ↑ knowledge in exposed population | p<0.001 | Large-scale program increased health knowledge alongside PA. |
| **Pentakota et al., 2019**[53] | BMI, Adequacy of PA (≥600 METs) | BMI not reported; Adequacy improved: Insufficient PA ↓ 18.6%→9.1%, Recommended ↑ 81.4%→90.9% | No control | Pre–post analysis showed improved adequacy of PA, but no weight/BMI data | p<0.001 | Smartphone app increased adequacy of PA, but no anthropometric measures reported. |
| **Mathews E, 2021**[55] | BMI, Waist Circumference | BMI: 26.9→26.9 (4 mo), 27.8 (13 mo); WC: 89.8→89.6 (4 mo), 89.2 (13 mo) | BMI: 26.7→26.9 (4 & 13 mo); WC: 87.9→88.0 (4 & 13 mo) | No significant between-group differences | NS | Peer-support program improved PA early, but no impact on BMI or waist circumference. |
| **Baldovino-Chiquillo L, 2023**[50] | BMI, Weight status | Walking ↑, but BMI/weight stable; small mixed park effects | Similar stability | No significant BMI or anthropometric change | NS | Urban renewal + cable-car program ↑ walking, but no anthropometric benefits. |
